# HSPA5 induces autophagy targeting VP2 through the PERK-eIF2α signaling pathway to inhibit SVA replication

**DOI:** 10.1128/jvi.02103-25

**Published:** 2026-04-13

**Authors:** Liang Li, Ruimin Bi, Jie Li, Meifang Chang, Jinting Zhao, Xuan Wang, Li Wei, Xinyue Chang, Yan Cheng, Zongjun Yin, Jun Wang, Yingchun Zhou, Zongyi Bo, Haixiao Shen, Junfang Yan, Xuelan Liu, Pei Sun

**Affiliations:** 1 College of Veterinary Medicine, Anhui Agricultural University 605541 , Hefei, Anhui, China; 2 Joint Research Center for Food Nutrition and Health of IHM, Anhui Agricultural University 12486, Hefei, China; 3 College of Animal Science and Technology, Anhui Agricultural University 12486, Hefei, China; 4 Anhui Provincial Center for Disease Control and Prevention 117844, Hefei, China; 5 Joint International Research Laboratory of Agriculture and Agri-Product Safety, The Ministry of Education of China, Yangzhou University 38043, Yangzhou, China; 6 College of Veterinary Medicine, Nanjing Agricultural University 70578, Nanjing, China; 7 Key Laboratory of Applied Technology on Green-Eco-Healthy Animal Husbandry of Zhejiang Province, Zhejiang Provincial Engineering Laboratory for Animal Health Inspection and Internet Technology, College of Animal Science and Technology, College of Veterinary Medicine, Zhejiang A&F University 12627, Lin'an, Zhejiang, China; University of Kentucky College of Medicine, Lexington, Kentucky, USA

**Keywords:** SVA, HSPA5, VP2, UPR, autophagy

## Abstract

Senecavirus A (SVA), also known as Seneca Valley virus (SVV), is an important small RNA virus that causes swine vesicular disease, posing a significant threat to the global swine industry. To date, the molecular mechanism of SVA replication remains poorly understood. Heat shock proteins (HSPs) are a family of molecular chaperone proteins that are widely present in organisms. More and more studies have shown that HSPs are associated with viral infections. Here, we screened HSPs and found that overexpression of HSPA5 significantly inhibited SVA replication, while knockout of HSPA5 promoted SVA proliferation. Further analysis showed that HSPA5 started to inhibit SVA proliferation at the stage of viral replication, and by screening SVA-encoded proteins, we found that HSPA5 degraded and interacted with the viral protein VP2 through the autophagy pathway. Mechanistically, HSPA5 participates in the degradation of VP2 proteins by activating autophagy mediated by the PERK-eIF2α pathway. In addition, the NBD region of HSPA5 is critical for VP2 degradation, and the Thr180 residue of VP2 has been identified as a key target for HSPA5 to inhibit the VP2 protein. The VP2 mutant virus rT180N showed resistance to the antiviral activity of HSPA5 compared with wild-type SVA and repair virus rT180N(R). In conclusion, these findings indicate that HSPA5 is an effective antiviral factor that suppresses SVA infection and contributes to a better understanding of the antiviral mechanism of HSPA5 and virus-host interactions.

**IMPORTANCE:**

Senecavirus A (SVA) infections have been reported in many pork-producing countries, but the lack of a commercial vaccine has caused significant economic losses to the world pig industry. In this study, we determined the antiviral role of HSPA5 in SVA and found that HSPA5 induces autophagy targeting VP2 through the PERK-eIF2α signaling pathway to inhibit SVA replication. In addition, the NBD region of HSPA5 is essential for the degradation of the VP2 protein, and HSPA5 targets the threonine residue at position 180 of the VP2 protein to exert its degradative function. These findings broaden the antiviral spectrum of SVA and provide a better understanding of the antiviral mechanism of SVA and virus-host interactions, providing important clues for the development of effective vaccines.

## INTRODUCTION

SVA is an emerging pathogen that causes swine vesicular disease and acute mortality in newborn piglets (1). In recent years, SVA has spread rapidly worldwide, especially in countries such as Brazil, China, and the United States of America (2–4). Taxonomically, SVA is classified in the genus Senecavirus in the family Picornaviridae. The genome of this virus is a linear, non-fragmented, single-stranded sense RNA, approximately 7.2 kb in length, with a large open reading frame (ORF) encoding a single polyprotein that is initially processed together with three precursor proteins (P1, P2, and P3) to form a leader protein (L). Subsequently, these three precursor proteins are cleaved to produce four structural proteins (VP1, VP2, VP3, and VP4) and seven non-structural proteins (2A, 2B, 2C, 3A, 3B, 3C, and 3D) (5). The VP2 protein contains multiple epitopes that specifically recognize and bind to receptors on the surface of the host cells. Due to its immunogenicity, VP2 is a major target for the elicitation of specific antibodies, especially neutralizing antibodies. Given its unique antigenic properties, VP2 has become an excellent candidate for the development of SVA vaccines (6). Currently, no commercial vaccines targeting SVA have emerged, posing significant challenges for the prevention and control of this virus (7).

Heat shock proteins (HSPs) are a highly conserved protein family widely present in living organisms. Their main function is to act as molecular chaperones, participating in protein folding, assembly, transport, and degradation to maintain cellular protein homeostasis (8). HSPs can be divided into several families based on their molecular weight, including HSP100, HSP90, HSP70, HSP60, HSP40, and small-molecule HSPs (HSP27) (9). In recent years, the HSP family has been demonstrated to participate in viral infection processes. These proteins can engage in the host’s defense mechanisms against exogenous pathogen invasion while also potentially being exploited by viruses to promote their replication and survival. For example, DNAJB6, a member of HSP40, significantly inhibits viral replication by interacting with the non-structural protein NS3 of Japanese encephalitis virus (JEV) (10). HSPA1L inhibits influenza A virus (IAV) replication by promoting the ubiquitination and autophagic degradation of viral neuraminidase (NA) (11). HSP90 enhances viral replication efficiency by stabilizing the large protein (L protein) of the measles virus (MuV), thereby promoting the formation of the viral polymerase complex (12). Given that the heat shock protein family influences viral replication and infection processes through interactions with viral proteins, regulation of host cell autophagy and ubiquitination processes, and stabilization of viral replication complexes, the biological functions of these family proteins warrant further exploration.

GRP78/BiP is a protein encoded by the HSPA5 gene, belonging to the heat shock protein family and serving as a key molecular chaperone in the endoplasmic reticulum (ER). It plays an essential role in protein folding and quality control (13, 14). The structure of GRP78/BiP comprises two primary domains: the nucleotide-binding domain (NBD) located in the N-terminal region and the substrate-binding domain (SBD) situated in the C-terminal region (15, 16). When unfolded or misfolded proteins accumulate in the ER beyond its capacity for effective folding, HSPA5 can trigger the unfolded protein response (UPR). This response serves to reduce the burden of unfolded or misfolded proteins, promote autophagy, and engage with apoptotic pathways to aid in determining cell survival (16–18). In recent years, it has been found that HSPA5 plays an important role in the replication of several viruses. It has been shown that HSPA5 is directly or indirectly involved in viral replication through interactions with viral proteins or host cell factors. For example, HSPA5 enhances viral replication by interacting with the dengue virus (DENV) nonstructural protein NS1, which stabilizes viral proteins and prevents them from being degraded by the proteasome (19). GRP78 (HSPA5) activates the NF-κB signaling pathway by interacting with the enterovirus-encoded RNA-dependent RNA polymerase (RdRp) 3D protein. This activation leads to the production of inflammatory factors that broadly inhibit enterovirus replication (20). However, the role of HSPA5 in SVA infection remains a mystery.

The UPR constitutes a critical signaling network in eukaryotic cells that responds to ER stress. This system dynamically regulates the protein folding capacity of cells by sensing the accumulation of unfolded or misfolded proteins within the ER, thereby maintaining cellular homeostasis (21). In mammalian cells, the UPR comprises three distinct pathways initiated by specific ER sensors: inositol-requiring enzyme 1 (IRE1), protein kinase RNA (PKR)-like ER kinase (PERK), and activating transcription factor 6 (ATF6) (22). When proteins fail to achieve their functional folded state, they are retained in the ER lumen, where terminally misfolded proteins can be degraded through autophagy (23). UPR-induced autophagy generally exerts a cytoprotective effect, further supporting its role in maintaining cellular homeostasis. HSPA5, a core regulator of the UPR, affects viral replication during viral infection by modulating the UPR signaling pathway. It has been found that Nsp2, a nonstructural protein of porcine reproductive and respiratory syndrome virus (PRRSV), interacts with HSPA5 to regulate the PERK and ATF6 branches of the UPR, thereby enhancing viral RNA synthesis and replication (24). Zika virus (ZIKV) infection induces ER stress, which leads to the upregulation of the expression of HSPA5, which facilitates folding of viral proteins and replication of viral RNA through the modulation of the UPR signaling pathway (25). Interestingly, this study reveals that HSPA5 inhibits SVA replication by activating the PERK branch of the UPR and interacting with the VP2 protein of SVA to mediate autophagic degradation of the VP2 protein. Therefore, this study expands the antiviral spectrum of HSPA5, particularly by targeting viral protein degradation through autophagy mediated by the UPR-PERK axis. It also provides significant insights for identifying antiviral targets and elucidating the pathogenesis of SVA infection.

## RESULTS

### Screening for HSPs that regulate SVA proliferation

**Fig 1:**
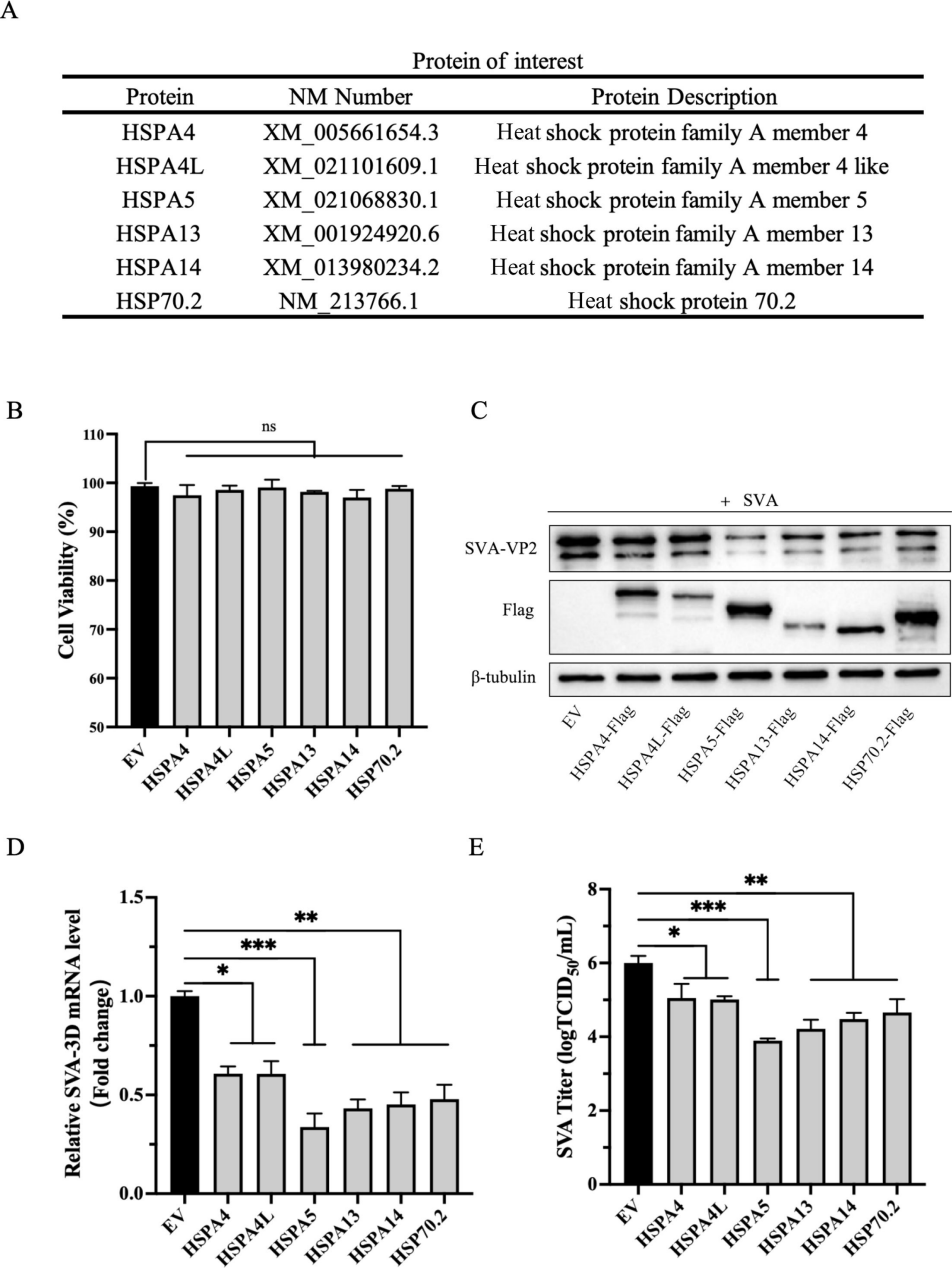
Screening for HSPs that regulate SVA proliferation. (**A**) Porcine HSPs family screening list. (**B**) Cell viability in cells overexpressing a specified gene (EV, empty vector, and pCAGGS). (**C**) BHK-21 cells were transfected with the specified genes, and after infection with SVA (0.01 MOI) for 16 h, the replication of SVA was assessed by western blotting. (**D**) Viral RNA was evaluated using RT-qPCR. (**E**) The supernatant was used to measure viral titers by TCID_50_ analysis. These results are from one of three independent experiments. Error bars indicate SD. Asterisks in the figure indicate significant differences (*P < 0.05; **P < 0.01; ***P < 0.001; ns: not significant).

To assess the potential impact of various HSPs on SVA infection, we selected a subset of HSP family genes (Fig. 1A), and the corresponding genes were cloned into the pCAGGS vector. The constructed recombinants were introduced into BHK-21 cells, and the cell viability assay showed uniform cell growth and activity in all transfected groups (Fig. 1B). After transfection, cells were inoculated with 0.01 MOI of SVA, and protein samples were collected 16 h later for western blot analysis. The results showed that all seven HSP recombinant proteins were successfully expressed, among which HSPA5 overexpression significantly inhibited the proliferation of SVA in BHK-21 cells (Fig. 1C). Meanwhile, the results obtained by viral genome as well as the viral titers detected by TICD_50_ were the same as those detected by protein blotting (Fig. 1D and E).

### HSPA5 overexpression inhibits SVA replication in a dose-dependent manner

**Fig 2:**
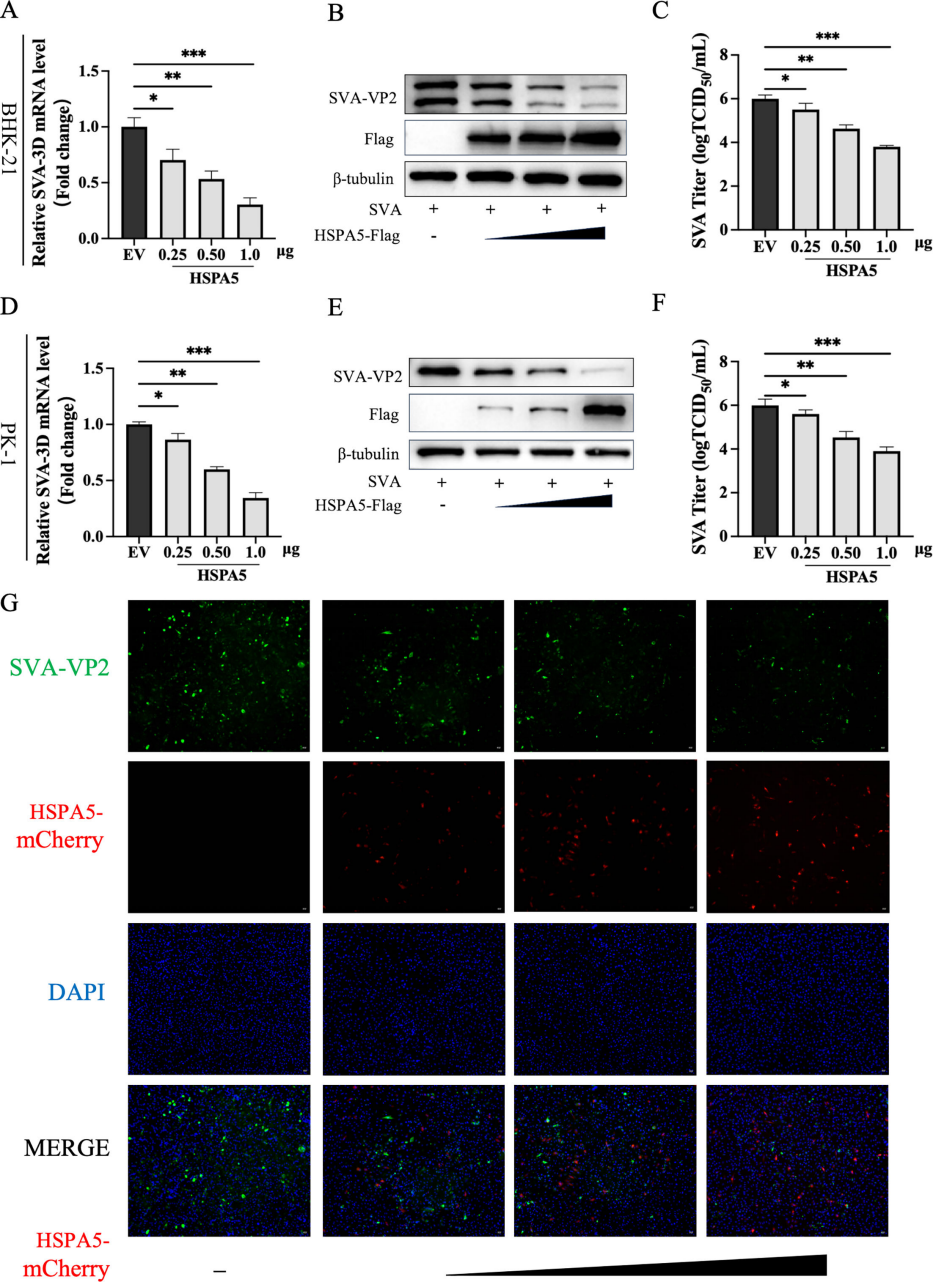
HSPA5 overexpression inhibits SVA replication in a dose-dependent manner. (**A**) BHK-21 cells were transfected with different doses of pCA-HSPA5-Flag plasmid, and after infection with SVA (0.01 MOI) for 16 h, Viral RNA was evaluated using RT-qPCR. (**B**) The replication of SVA was assessed by western blotting. (**C**) The supernatant was used to measure viral titers by TCID_50_ analysis. (**D**) PK-1 cells were treated the same as BHK-21 cells, and the antiviral effect of HSPA5 was detected by RT-qPCR. (**E**) The replication of SVA was assessed by western blotting. (**F**) The supernatant was used to measure viral titers by TCID_50_ analysis. (**G**) IFA images showing viral VP2 protein in green, HSPA5 protein in red, and nuclei in blue. These results are from one of three independent experiments. Error bars indicate SD. Asterisks in the figure indicate significant differences (*P < 0.05; **P < 0.01; ***P < 0.001).

To further clarify the role of HSPA5 in SVA proliferation, the porcine HSPA5 proteins were overexpressed in BHK-21 and PK-1 cells, respectively, for 24 h and then infected with SVA (0.01 MOI). Then, the cells and supernatants of the infected cell cultures were collected and used for the determination of SVA replication levels. As shown in Fig. 2A and D, viral transcription levels exhibited a dose-dependent downregulation with increasing transfection doses of the recombinant plasmid pCA-HSPA5-Flag compared to the pCAGGS (empty vector, EV) group. Concurrently, western blot analysis of viral protein expression (Fig. 2B and E) and TICD_50_ assays of viral titers (Fig. 2C and F) yielded results consistent with viral genome detection. Furthermore, indirect immunofluorescence assay (IFA) also detected a dose-dependent reduction in VP2 fluorescence intensity with increasing HSPA5 expression (Fig. 2G), confirming the dose-dependent antagonistic effect of HSPA5 against SVA.

### Knockout of HSPA5 enhances SVA replication

**Fig 3:**
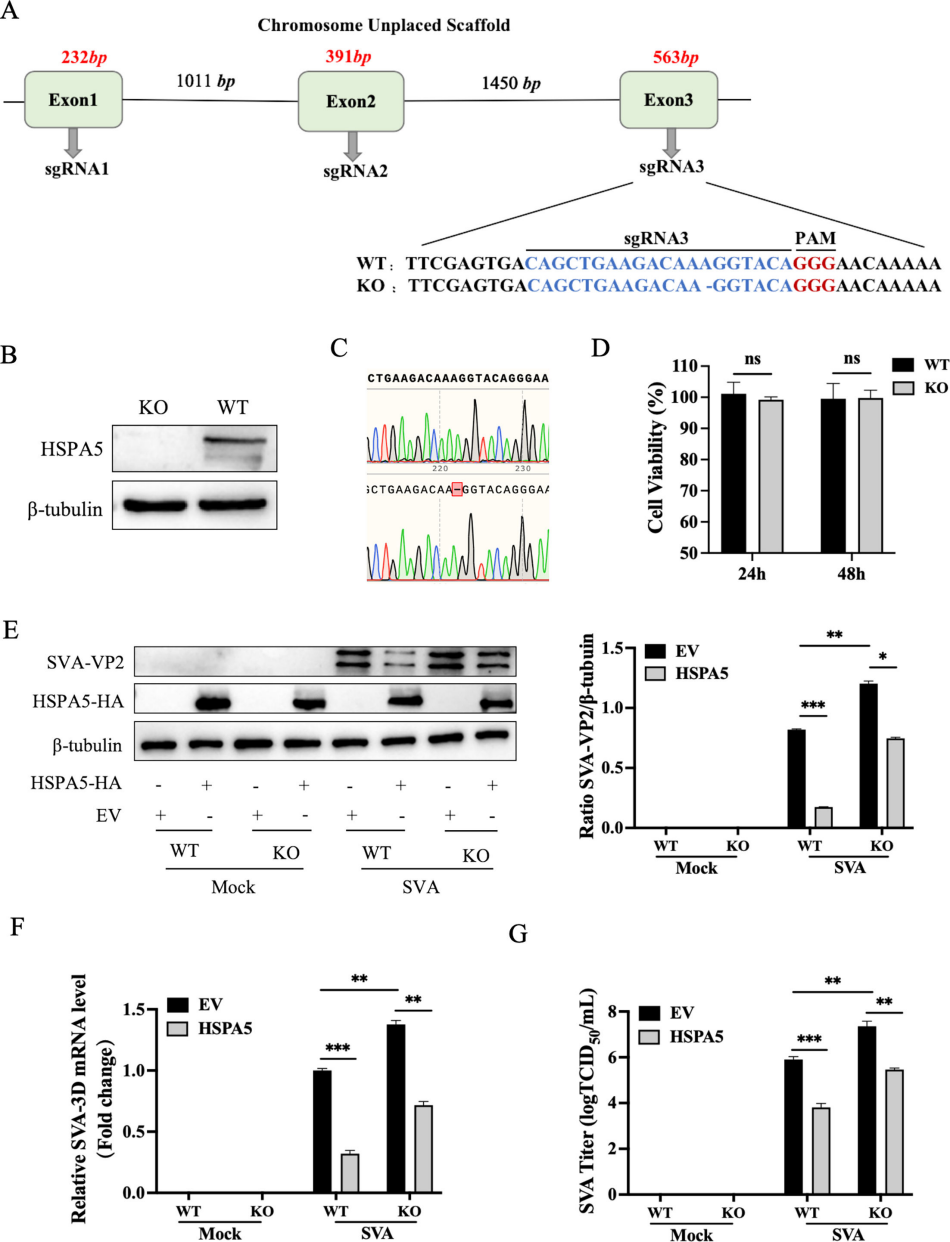
Knockout of HSPA5 enhances SVA replication. (**A**) Sequences of HSPA5 targeted by sgRNAs 1–3. The blue short line indicates a missing base. GGG is the protospacer adjacent motif (PAM). (**B**) Western blot analysis of HSPA5 expression in the BHK-HSPA5-KO and BHK-Wt cells. (**C**) Sanger sequencing revealed the missing in HSPA5. (**D**) Viability assay of BHK-Wt and BHK-HSPA5-KO cells. Cells were seeded into wells of 96-well cell culture plates, and viability was measured at 24 h and 48 h. (**E-G**) BHK-HSPA5-KO and BHK-Wt cells were transfected with HSPA5-HA or empty vector and then challenged with SVA (0.01 MOI) for 16 h. VP2 protein levels, 3D mRNA levels, and viral titers were evaluated by western blotting, RT-qPCR, and TCID_50_, respectively. These results are from one of three independent experiments. Error bars indicate SD. Asterisks in the figure indicate significant differences (*P < 0.05; **P < 0.01; ***P < 0.001; ns: not significant).

The role of HSPA5 in SVA replication was further investigated using HSPA5 knockout BHK-21 cells (BHK-HSPA5-KO) (Fig. 3A). Western blotting and sequencing results confirmed that the HSPA5 gene has been disrupted successfully (Fig. 3B and C). There was no significant difference in cell viability between BHK-21 and BHK-HSPA5-KO (Fig. 3D). Subsequently, the replication efficiency of SVA was measured in BHK-HSPA5-KO and BHK-Wt cells. SVA replication levels in cells were significantly promoted compared to WT as determined by western blotting for VP2 (Fig. 3E), RT-qPCR (Fig. 3F), and titers (Fig. 3G). These results indicate that HSPA5 is crucial for SVA replication.

### HSPA5 exerts an inhibitory effect during viral replication

**Fig 4:**
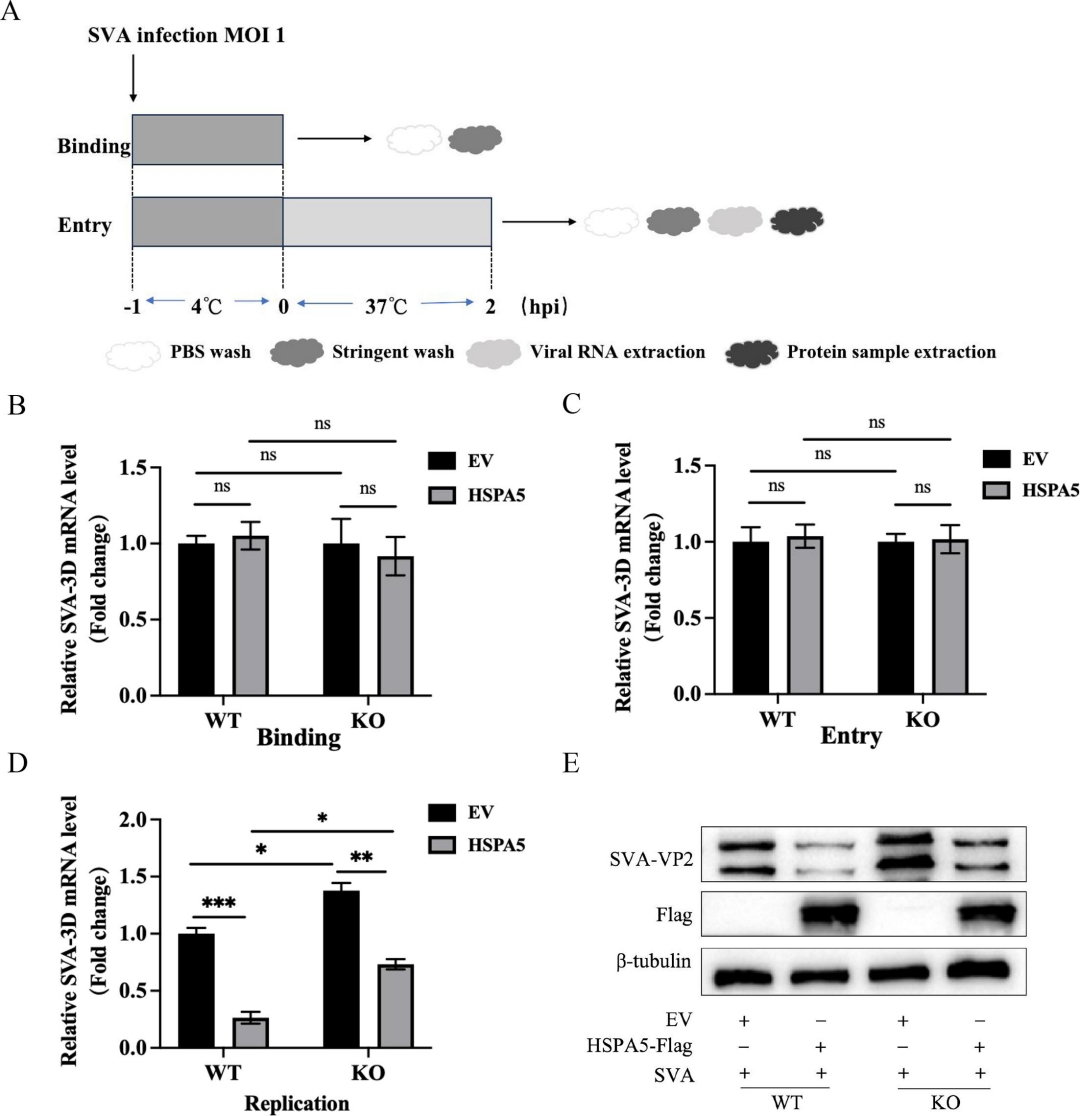
The effects of HSPA5 on the SVA life cycle. (**A**) Overview of the experimental design to examine virus binding and entry. (**B and C**) RT-qPCR results for viral mRNA (0 and 2 hpi) in pCA-HSPA5-Flag or empty vector-transfected BHK-21 cells and BHK-HSPA5-KO cells challenged with SVA. (**D**) RT-qPCR results for viral mRNA (8 hpi) in pCA-HSPA5-Flag or empty vector-transfected BHK-21 cells and BHK-HSPA5-KO cells. (**E**) Western blotting was used to detect SVA-VP2 protein levels in BHK-21 cells transfected with pCA-HSPA5-Flag or empty vector, as well as in BHK-HSPA5-KO cells (8 hpi). These results are obtained from one of three independent experiments. Error bars indicate SD. Asterisks in the figure indicate significant differences (**P < 0.01; ***P < 0.001; ns: not significant).

To further investigate the mechanism by which HSPA5 inhibits SVA infection, this study examined the effects of HSPA5 on the SVA life cycle in BHK-21 cells and BHK-HSPA5-KO cells. We first analyzed the effects of HSPA5 on SVA adsorption and internalization via RT-qPCR (Fig. 4A). The results showed that SVA 3D mRNA levels in HSPA5-treated cells were similar to those in EV-treated cells, indicating that HSPA5 does not block the binding and the entry of SVA (Fig. 4B and C). Subsequently, we examined the effect of HSPA5 on SVA replication. The results indicate that HSPA5 interferes with viral replication (Fig. 4D). Concurrently, western blot analysis confirmed findings consistent with viral transcription levels (Fig. 4E). Collectively, these results demonstrate that HSPA5 inhibits SVA during the replication stage.

### HSPA5 degrades SVA-VP2 protein and interacts with it

**Fig 5:**
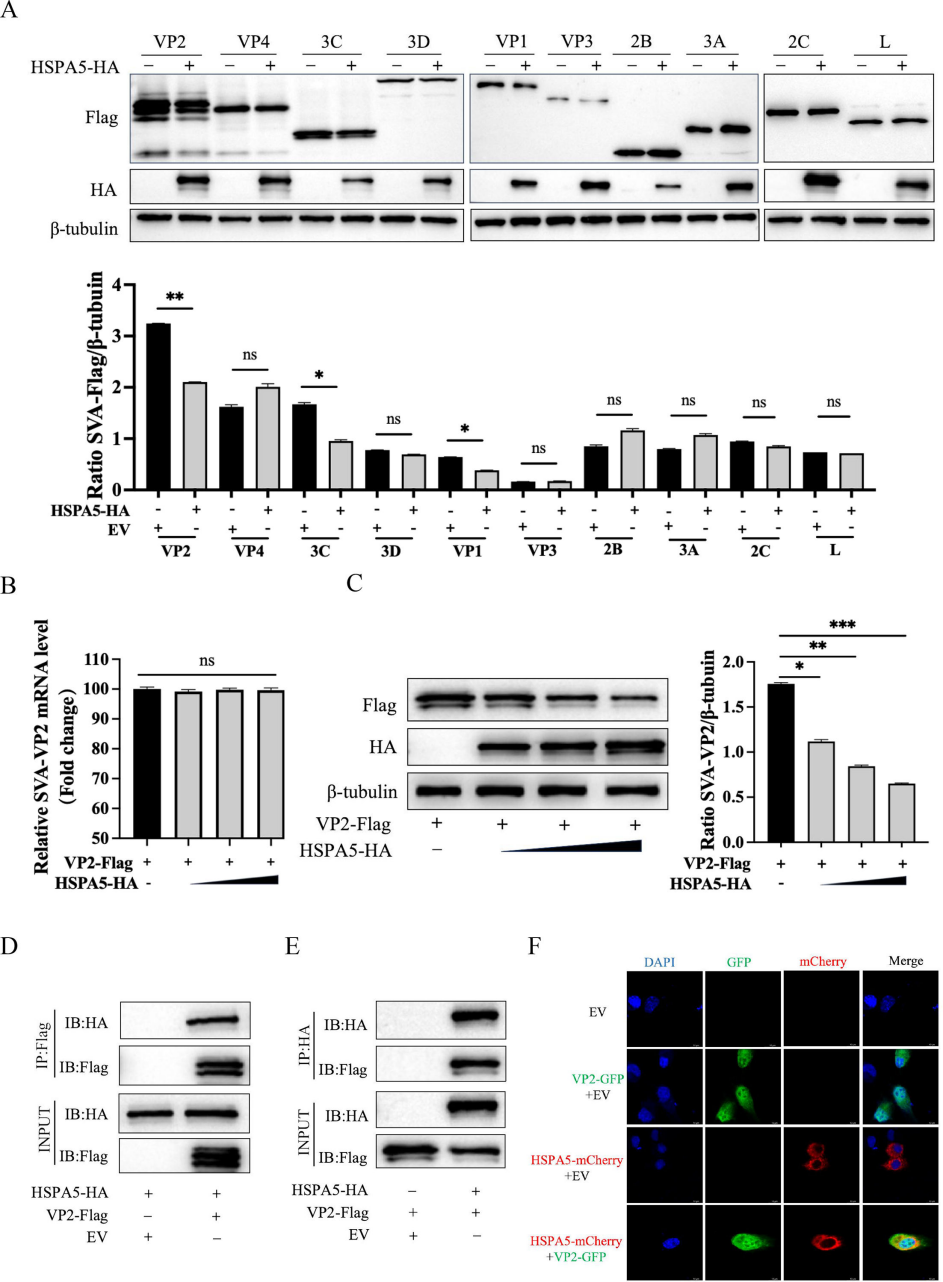
HSPA5 degrades SVA-VP2 protein and interacts with it. (**A**) HEK-293T cells were transfected with the indicated plasmids. After incubation for 24 h, western blotting assays were performed to determine the expression of pCA-HSPA5-HA and Flag-tagged viral proteins. (**B**) HEK-293T cells were transfected with pCA-VP2-Flag along with vector or different doses of pCA-HSPA5-HA plasmid; the transcription levels of VP2 were evaluated by RT-qPCR. (**C**) HEK-293T cells were transfected with pCA-VP2-Flag along with vector or different doses of pCA-HSPA5-HA plasmid, and the protein expression levels were evaluated by western blotting. (**D, E**) HEK-293T cells co-transfected with pCA-VP2-Flag and pCA-HSPA5-HA were subjected to Co-IP, analyzed by western blotting using anti-Flag and anti-HA antibodies. (**F**) Recombinant plasmid pCA-HSPA5-mCherry (red light) and pCA-VP2-GFP (green light) were co-transfected into BHK-21 cells and observed on a confocal laser microscope. These results are obtained from one of three independent experiments. Error bars indicate SD. Asterisks in the figure indicate significant differences (*P < 0.05; **P < 0.01; ***P < 0.001; ns: not significant).

It has been established that HSPA5 inhibits the replication stage of SVA. This study further explores the relationship between HSPA5 and viral proteins. pCAGGS-HSPA5-HA plasmid or vector plasmid and plasmids expressing Flag-tagged viral proteins (VP1, VP2, VP3, VP4, L, 2B, 2C, 3A, 3C, and 3D) were co-transfected into HEK-293T cells. Western blotting analysis revealed that HSPA5 degrades SVA VP1, VP2, and 3C proteins (Fig. 5A). Given the extensive research on the mechanism of the 3C protein, we will focus our future efforts on the VP2 protein. Subsequent results demonstrated that HSPA5 significantly degrades the viral VP2 protein in a dose-dependent manner without affecting VP2 transcription levels (Fig. 5B and C). Co-immunoprecipitation assay showed that HSPA5 interacted with VP2 (Fig. 5D and E). Finally, to visualize the subcellular localization of these interactions, we constructed GFP-labeled VP2 and mCherry-labeled HSPA5 plasmids, transfected them into BHK-21 cells, and observed them with confocal laser microscopy. Confocal imaging results revealed colocalization of HSPA5 and SVA-VP2 in the cytoplasm (Fig. 5F). To further validate the interaction between HSPA5 and SVA VP2 during SVA infection, BHK-21 cells overexpressing HSPA5 were infected with SVA and harvested 16 h post-infection. Immunoprecipitation was performed using an anti-VP2 antibody. The results demonstrated that overexpressed HSPA5 interacts with VP2 during SVA infection (Fig. S1A). Furthermore, confocal microscopy revealed that overexpressed HSPA5 colocalizes with VP2 in BHK-21 cells during SVA infection (Fig. S1C).

### HSPA5 induces autophagy and degrades VP2 through the autophagy-lysosome pathway

**Fig 6:**
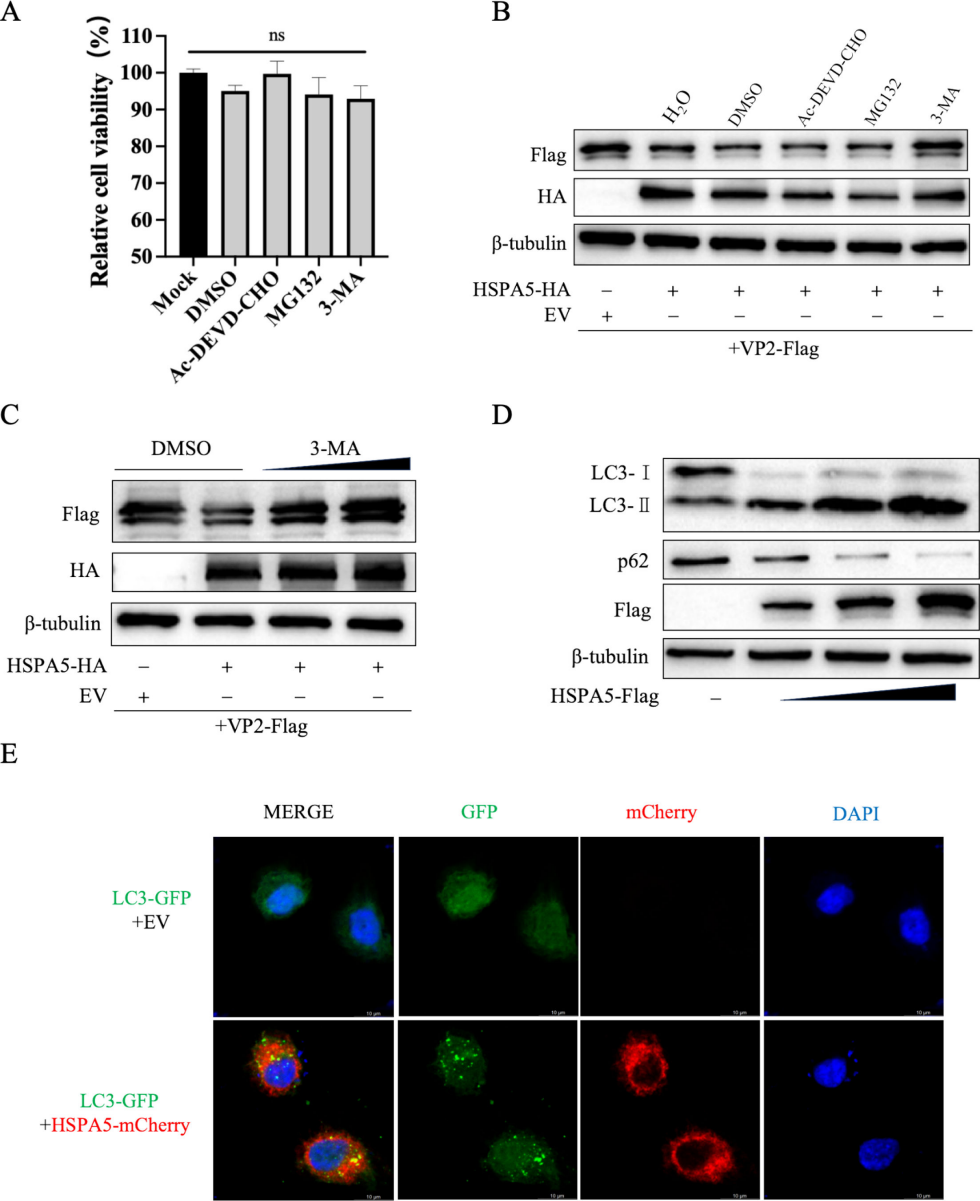
HSPA5 induces autophagy and degrades VP2 through the autophagy-lysosome pathway. (**A**) The effect of small molecule inhibitors on cell viability was detected using the CCK-8 cell viability assay reagent according to the manufacturer’s instructions. (**B**) HEK-293T cells were transfected with pCA-HSPA5-HA and pCA-VP2-Flag, and the cells were then treated with MG132 (10 μM), 3-methyladenine (3-MA; 50 μM), or Ac-DEVD-CHO (20 μM) 8 h post-transfection. (**C**) HEK-293T cells were transfected with HSPA5-HA and VP2-Flag, and the cells were then treated with different doses of 3-MA 8 h post-transfection. (**D**) HEK-293T cells were transfected with different doses of HSPA5-Flag plasmid and then collected for western blotting. (**E**) HEK-293T cells were transfected with LC3-GFP and HSPA5-mCherry plasmids. At 24 h post-transfection, the cell nuclei were stained with DAPI (blue) and analyzed by confocal microscopy. These results are from one of three independent experiments. Error bars indicate SD. Asterisks in the graph indicate significant differences (ns: not significant).

The apoptosis, autophagy, and ubiquitin-proteasome system are three major intracellular degradative pathways. To determine the degradation pathway of VP2 protein by HSPA5, we co-transfected recombinant plasmids pCA-HSPA5-HA and pCA-VP2-Flag into HEK-293T cells and then treated them with Ac-DEVD-CHO (inhibitor of the apoptosis pathway), 3-methyladenine (3-MA) (inhibitors of the autophagy pathway), and MG132 (inhibitor of the ubiquitin-proteasome pathway), respectively. Notably, cell viability assays confirmed that none of the inhibitors or the control reagent DMSO exhibited detectable cytotoxicity (Fig. 6A). The western blotting results showed that treatment with 3-MA can largely restore VP2 levels, whereas no obvious differences in VP2 levels were observed under the treatment with Ac-DEVD-CHO or MG132 as compared to the control dimethyl sulfoxide (DMSO) treatment (Fig. 6B), suggesting that the autophagy-lysosome pathway plays a major role in the degradation of VP2. In order to further determine the role of 3-MA, experiments were conducted using different doses of 3-MA, and it was found that the VP2 protein was rescued by 3-MA in a dose-dependent manner (Fig. 6C). As described above, HSPA5 degrades VP2 expression through the autophagic lysosomal pathway. We further hypothesized that HSPA5 may affect autophagosome formation. To determine whether HSPA5 caused autophagy, we analyzed changes in LC3-II protein expression in cells. We found that overexpression of HSPA5 significantly increased the conversion of endogenous LC3-I to LC3-II and that LC3-II increased significantly with increasing doses of transfected HSPA5 (Fig. 6D). Additionally, we simultaneously measured another important autophagy-related target, p62. As shown in Fig. 6D, p62 protein expression was gradually decreased in transfected HSPA5 cells. The results indicated that HSPA5 could induce autophagy. Meanwhile, an increase in LC3-GFP spot formation was clearly detected in cells overexpressing HSPA5 (Fig. 6E). Collectively, these results suggest that HSPA5 specifically mediates VP2 degradation, which is associated with the autophagy pathway.

### HSPA5 induces autophagy-mediated degradation of VP2 by activating the PERK pathway of the UPR

**Fig 7:**
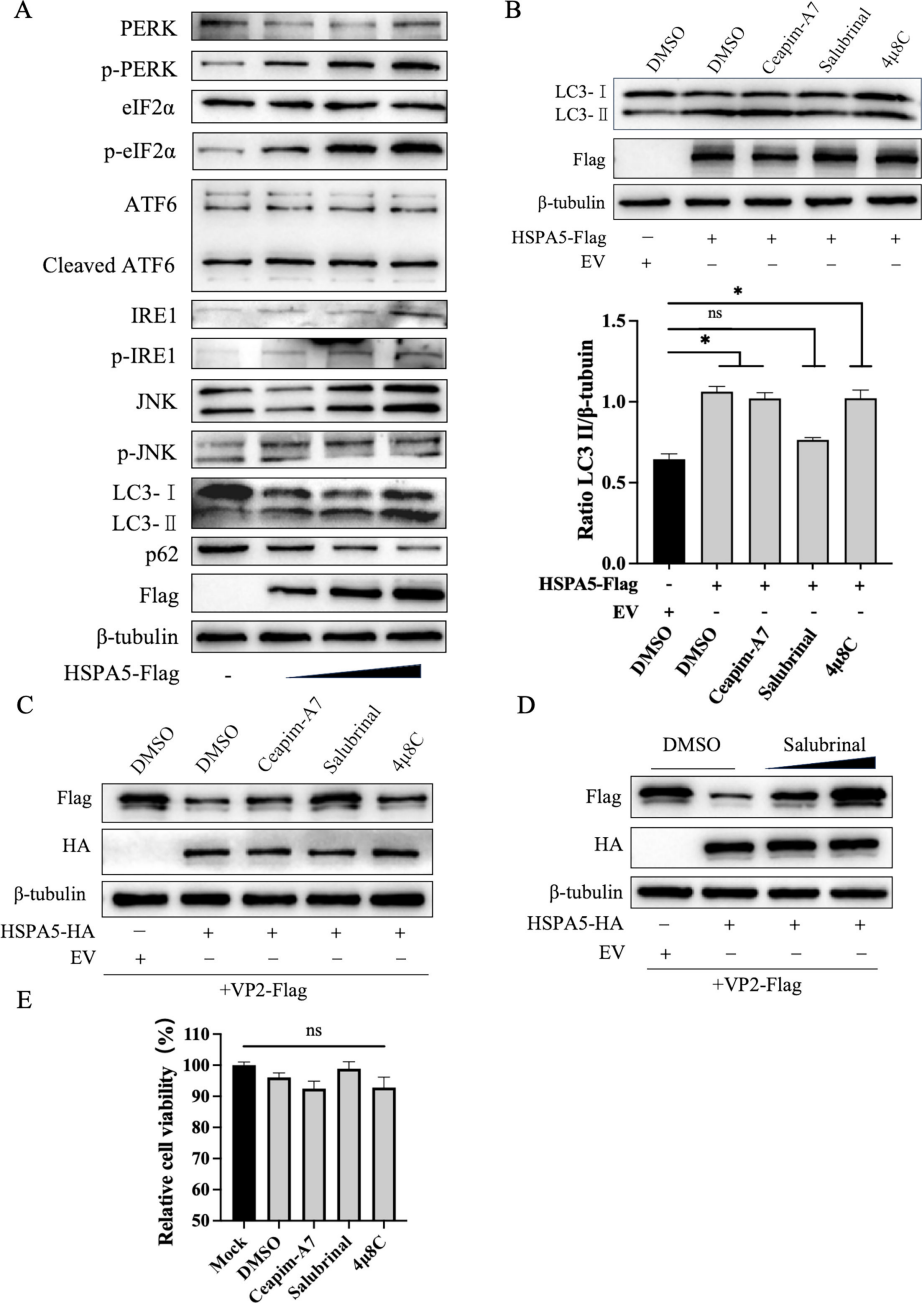
HSPA5 degrades VP2 by inducing autophagy through activating the PERK-mediated pathway of UPR. (**A**) Different doses of pCA-HSPA5-Flag were transfected into HEK-293T cells, and after 24 h, the cell lysates were collected and analyzed for the expression of PERK, IRE1, and ATF6 pathway proteins by western blotting. (**B**) HEK-293T cells were transfected with pCA-HSPA5-Flag, and the cells were then treated with Salubrinal (30 μM), 4μ8C (40 μM), or Ceapim-A7 (50 μM) at 8 h post-transfection, and the cells were then collected for western blotting assay at 16 h. (**C**) HEK-293T cells were transfected with pCA-HSPA5-HA and pCA-VP2-Flag, and the cells were treated with Salubrinal (30 μM), 4μ8C (40 μM), or Ceapim-A7 (50 μM) at 8 h post-transfection, and the cells were collected for western blotting assay 16 h later. (**D**) HEK-293T cells were transfected with pCA-HSPA5-HA and pCA-VP2-Flag, and the cells were then treated with different doses of Salubrinal 8 h post-transfection. (**E**) The effect of small-molecule inhibitors on cell viability was detected using the CCK-8 cell viability assay reagent according to the manufacturer’s instructions. These results are obtained from one of three independent experiments. Error bars indicate SD. Asterisks in the graph indicate significant differences (*P < 0.05; ns: not significant).

To determine whether HSPA5-induced autophagy originates from activation of the UPR signaling pathway, we analyzed the three branches of the UPR signaling network. Transfection of HEK-293T cells with varying doses of the recombinant plasmid pCA-HSPA5-Flag revealed, via western blotting analysis, that increased expression of phosphorylated PERK correlated with higher HSPA5 transfection levels. Concurrently, a significant increase in the expression of its downstream effector, phosphorylated eIF2α (p-eIF2α), was observed. Additionally, levels of phosphorylated IRE1 (p-IRE1) and phosphorylated JNK (p-JNK) were markedly elevated, and cleaved ATF6 (50 kDa protein) expression increased (Fig. 7A). We confirmed that HSPA5 activates all three branches of the UPR signaling pathway. Next, we validated which branch of the UPR signaling pathway is associated with autophagy induction. HEK-293T cells were transfected with the recombinant plasmid pCA-HSPA5-Flag. After 8 h, the cells were treated with the PERK inhibitor Salubrinal, the IRE1 inhibitor 4μ8C, and the ATF6 inhibitor Ceapim-A7 for 16 h. Cell lysates were collected, and western blotting was performed to detect changes in LC3 protein expression. The results showed that compared to the control group, the PERK inhibitor Salubrinal effectively suppressed the increase in LC3-II protein induced by HSPA5 (Fig. 7B). These findings indicate that HSPA5 induces autophagy via the PERK pathway, rather than the IRE and ATF6 pathways. To further determine whether HSPA5 degrades VP2 protein via the PERK pathway, recombinant plasmids pCA-HSPA5-HA and pCA-VP2-Flag were co-transfected into HEK-293T cells. Eight hours post-transfection, the cells were treated with the PERK inhibitor Salubrinal, the IRE1 inhibitor 4μ8C, and the ATF6 inhibitor Ceapim-A7 for 16 h. Cell lysates were collected, and VP2 protein expression levels were detected by western blotting. The results showed that VP2 protein levels were significantly restored in the Salubrinal-treated group compared to the control group, whereas no similar restoration was observed in the 4μ8C- or Ceapim-A7-treated groups (Fig. 7C). To further characterize Salubrinal’s mechanism, experiments were conducted using different concentrations of Salubrinal. The results demonstrated that Salubrinal rescued VP2 protein in a dose-dependent manner (Fig. 7D). Notably, cell viability assays confirmed that all inhibitors and the control reagent DMSO exhibited no detectable cytotoxicity (Fig. 7E). As described above, HSPA5 induces autophagy-mediated degradation of VP2 via the PERK pathway of the UPR.

### The NBD domain of HSPA5 targets the 180th amino acid residue of VP2 to exert its degradation function

**Fig 8:**
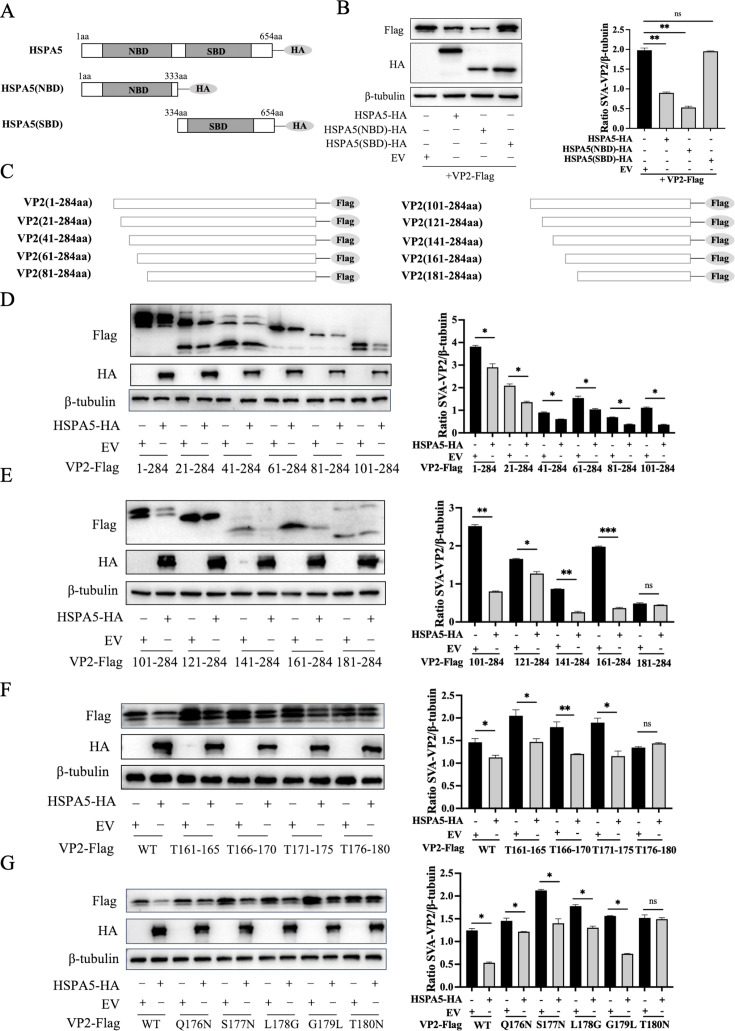
The NBD region of HSPA5 mediates its degradation function, and the 180th amino acid residue of VP2 is a key site for HSPA5 degradation. (**A**) HSPA5 truncated body construction strategy. (**B**) HEK-293T cells were cotransfected with pCA-VP2-Flag and HA-labeled HSPA5 or HSPA5 truncator, respectively, and the cells were lysed 24 h later. Samples were analyzed by western blotting. (**C**) VP2 truncated body construction strategy. (**D, E**) HEK-293T cells were co-transfected with pCA-HSPA5-HA and Flag-labeled VP2 or VP2 truncated bodies, respectively, and the cells were lysed 24 h later. Samples were analyzed by western blotting. (**F, G**) HEK-293T cells were co-transfected with pCA-HSPA5-HA and Flag-tagged VP2 or VP2 mutants, respectively, and the cells were lysed 24 h later. Samples were analyzed by western blotting. The mutants in F have alanine substitutions for the wild-type amino acids. These results are from one of three independent experiments. Error bars indicate SD. Asterisks in the graph indicate significant differences (*P < 0.05; **P < 0.01; ***P < 0.001; ns: not significant).

In order to determine the domain of HSPA5 responsible for VP2 degradation, we constructed HA-tagged HSPA5 truncated variants, including HSPA5 (NBD) (aa 1–333) and HSPA5 (SBD) (aa 334–652) (Fig. 8A). Co-transfect these truncated forms with Flag-tagged VP2 plasmids into HEK-293T cells. Cells were collected 24 h after transfection, and the degradation of VP2 was analyzed by western blotting. The experimental results indicate that HSPA5 (NBD) exhibits significant degradation of VP2, and HSPA5 (SBD) does not exhibit degradation (Fig. 8B). These results suggest that the NBD region of HSPA5 is the key structural domain that exerts its degradation function.

Subsequently, to explore the critical site where VP2 is degraded by HSPA5, nine Flag-tagged VP2 truncation variants were constructed by generating consecutive amino acid deletions in 20-residue increments. These included variants spanning aa 21–284, 41–284, 61–284, 81–284, 101–284, 121–284, 141–284, 161–284, and 181–284 (Fig. 8C). Flag-tagged VP2 and its truncated fragments were co-transfected with HA-tagged HSPA5 into HEK-293T cells. Cell samples were collected 24 h later for western blot analysis. The results showed that the expression level of VP2 aa 181–284 was restored (Fig. 8D and E), indicating that the critical site for VP2 degradation is located in the aa 161–180 region. Key residues in the aa 161–180 region were mapped using alanine scanning (26), and the following four Flag-tagged VP2 mutants were constructed: VP2 (T161–165), VP2 (T166–170), VP2 (T171–175), and VP2 (T176–180). Similarly, co-transfection of these mutants with HSPA5 restored VP2 (T176–180) expression levels (Fig. 8F), confirming that the key site is located within the 176–180 amino acid region. By identifying individual amino acid residues within this region, five point mutations were introduced at the following five distinct positions: VP2Q176N, VP2S177N, VP2L178G, VP2G179L, and VP2T180N. Subsequent validation showed that VP2T180N restored expression at the protein level (Fig. 8G), indicating that the functional site responsible for the targeted degradation of VP2 is located at threonine 180.

### Recombinant viruses harboring the T180N mutation exhibit resistance to the antiviral activity of HSPA5

**Fig 9:**
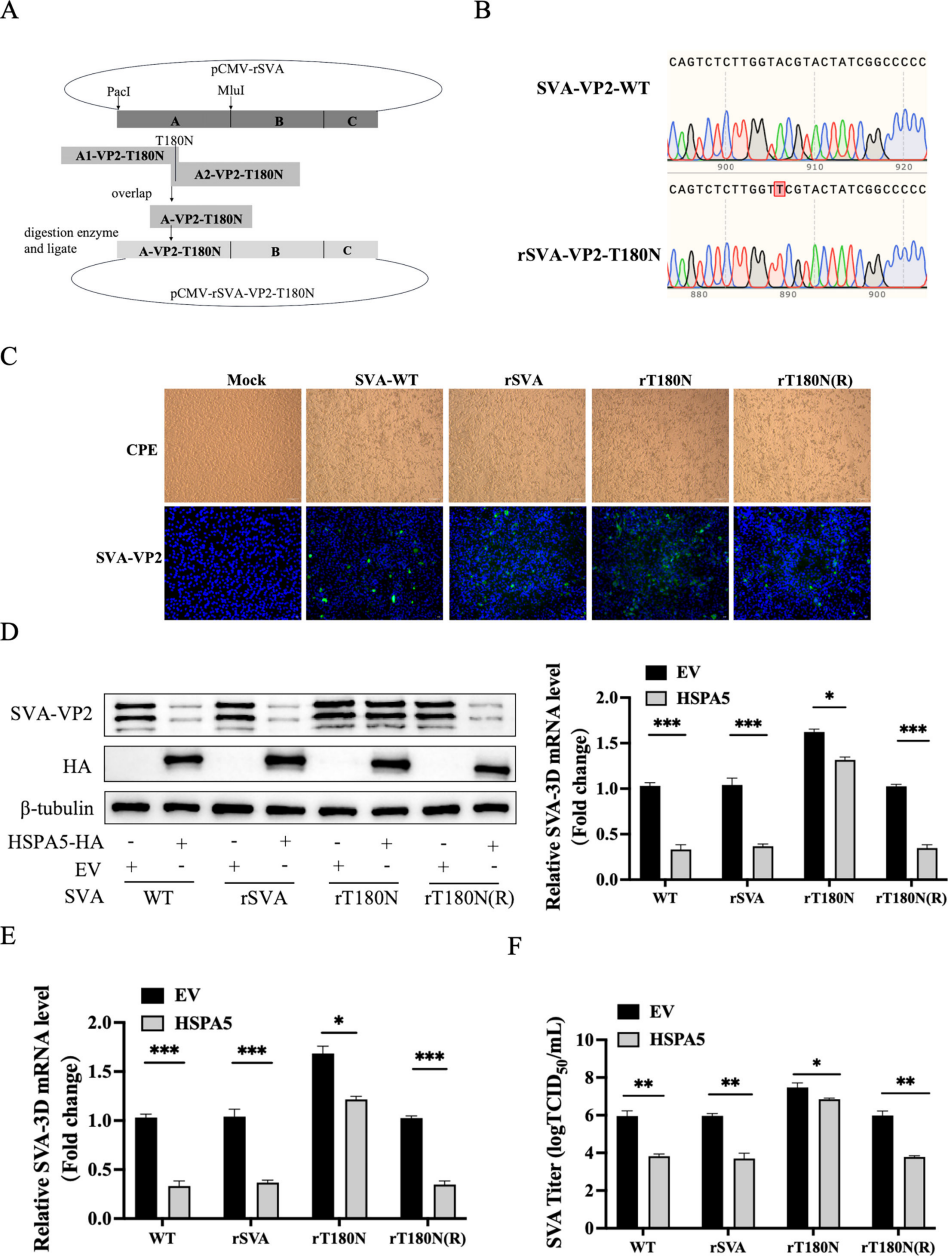
Resistance of recombinant virus with T180N mutation to HSPA5 antiviral activity. (**A**) A strategy of building recombinant SVA with a mutation at T180N of VP2. (**B**) Sequencing result of infectious cloning plasmids with a mutation at T180N of VP2. (**C**) The mutant infectious cloning plasmid was transfected into BHK-21 cells, causing cytopathy in BHK-21 cells. The infected cloning plasmid was transfected into BHK-21 cells, and the wild-type SVA was used as a positive control, and the cytopathy was observed after plasmid transfection for 36 h. The virus was collected, BHK-21 cells were infected with SVAs [WT, rSVA, rT180N, or rT180N (R)] for 16 h, and immunofluorescence identification was performed using SVA-VP2 polyclonal antibody. (**D**) BHK-21 cells were transfected with pCA-HSPA5-HA or empty vector for 24 h and then infected with SVAs [WT, rSVA, rT180N, or rT180N (R)] (0.01 MOI ) for 16 h. Cell samples were harvested and subjected to western blotting. (**E**) RT-qPCR. (**F**) TCID_50_. These results are from one of three independent experiments. Error bars indicate SD. Asterisks in the graph indicate significant differences. (**P < 0.01; ***P < 0.001; ns: not significant).

To determine the importance of VP2 Thr180 in SVA replication capacity, we first constructed the VP2 Thr180 mutation recombinant plasmid pCMV rSVA-VP2-T180N in BHK-21 cells using reverse genetics (Fig. 9A and B). Using the pCMV rSVA-VP2-T180N mutant recombinant plasmid as a foundation, we obtained the pCMV rSVA-VP2-T180N (R) mutant recovery recombinant plasmid. SVA-WT, rSVA, rT180N, and rT180N (R) were inoculated into BHK-21 cells, and immunofluorescence analysis was performed using SVA-VP2 polyclonal antibody after 16 h. The results showed that rT180N and rT180N (R) exhibited specific green fluorescence compared to the negative control, indicating successful rescue of these viruses (Fig. 9C). To further compare the replication capabilities of SVA-WT, rSVA, rT180N, and rT180N (R), BHK-21 cells were transfected with either HSPA5-HA or an empty vector. Twenty-four hours later, the four viruses were inoculated into BHK-21 cells at an MOI of 0.01. Sixteen hours post-inoculation, western blotting, RT-qPCR, and TCID_50_ assays revealed that HSPA5 significantly inhibited the replication of SVA-WT, rSVA, and rT180N(R). However, the inhibitory effect on rT180N was markedly reduced (Fig. 9D through F). These results confirm that rSVA-VP2-T180N confers resistance to HSPA5-mediated antiviral activity and identify the threonine residue at position 180 of the SVA-VP2 protein as a critical site for HSPA5-mediated inhibition of SVA replication.

## DISCUSSION

SVA is one of the most important pathogens affecting the swine industry. Until now, the underlying mechanisms by which SVA viral proteins interact with host cell proteins have not been fully understood. Therefore, understanding the interactions between SVA and its host not only elucidates the viral infection process within host cells but also aids in identifying antiviral factors and revealing their specific antiviral mechanisms. In recent years, studies have revealed that the heat shock protein family plays a crucial role in regulating the replication of various viruses, including JEV (10), infectious bronchitis virus (IBV) (27), foot-and-mouth disease virus (FMDV) (28), and hepatitis C virus (HCV) (29), among others. Recent studies confirm that HSP70 primarily enhances SVA replication through interactions between its SBD domain and viral L and 3D proteins (30). Additionally, another study demonstrated that STUB1 directly interacts with the viral protein VP1, mediating its ubiquitin-dependent degradation and thereby significantly inhibiting viral replication. In this process, HSP70 and HSC70 promote STUB1-mediated VP1 degradation by enhancing their interaction (31). Given the crucial role this family plays in viral regulation, we screened some members of the HSP protein family and found that HSPA5 effectively inhibited SVA replication in BHK-21 cells. Interestingly, two distinct proteins from the same family, HSP70.2 (NM213766.1) and HSP70 (NM_001123127) (30), exert opposite effects on SVA replication. This seemingly contradictory phenomenon precisely reveals the intricate functional specialization and high complexity within the HSP70 protein family during virus-host interactions.

Our results demonstrate that overexpression/knockout of HSPA5 significantly regulates SVA proliferation. Furthermore, previous studies have confirmed that HSPA5 can inhibit the replication of hepatitis B virus (HBV) and ZIKV (32, 33). These findings indicate that the HSPA5 protein may function as an antiviral factor to inhibit SVA replication, warranting further investigation. As is well known, the viral life cycle is the central process of viral proliferation, and its key steps include attachment, entry, replication, and release of viral particles. These steps not only determine the replication efficiency of the virus but also affect its pathogenicity and transmissibility (34). Therefore, exploring the effect of HSPA5 on the life cycle of SVA is of great interest to our understanding of the molecular mechanisms of HSPA5-mediated regulation of viral replication. Previous studies predominantly employed multi-step growth conditions to assess the impact of host proteins on different stages of the viral life cycle (35). Our results indicate that HSPA5 does not block the process of viral binding or entry, but HSPA5 initiates the inhibition of SVA proliferation during the replication phase of SVA. Other studies have investigated specific stages of the viral life cycle using inhibitors (30). Therefore, we simultaneously utilized an HSPA5 inhibitor to assess its impact on the SVA life cycle. Consistent with the above findings, SVA infection during the binding and entry stages did not depend on HSPA5, suggesting that HSPA5 primarily participates in the post-entry phase of the SVA life cycle (Fig. S7A through D). HSPA5 plays a significant role in the replication of multiple viruses. For instance, during IAV infection, HSPA5 enhances viral attachment and internalization by interacting with the viral hemagglutinin (HA) protein, thereby promoting viral replication (36). Similarly, in Newcastle disease virus (NDV) infection, HSPA5 directly binds to the viral HN protein, enhancing viral adsorption and replication within cells (37). Furthermore, HSPA5 is critical in Ebola virus (EBOV) infection, where the virus exploits HSPA5 as a host factor to promote replication and infection (38). Our findings indicate that HSPA5 can target VP2 for degradation and interact with it. Confocal microscopy showed that HSPA5 and VP2 can colocalize in the cytoplasm. HSPA5 also colocalizes and interacts with the viral VP2 protein in SVA-infected BHK-21 cells, indicating a correlation between HSPA5 inhibition of virus replication and its targeted degradation of VP2 protein.

Cystatin, ubiquitin-proteasome, and autophagy-lysosomal pathways are the three major systems that control protein degradation in eukaryotic cells (39–41). In the present study, we found that the autophagy/lysosomal inhibitor 3-MA inhibited HSPA5-mediated VP2 degradation, but treatment with the proteasome inhibitor MG132 and the caspase inhibitor Ac-DEVD-CHO failed to alleviate VP2 degradation. Consistent with this, OASL inhibits viral replication against infectious bursal disease virus (IBDV) by binding to the viral protein VP2 and targeting it to the autophagic pathway for degradation (42). Similarly, SQSTM1/p62, a selective autophagy receptor, is able to bind to the VP1 and VP3 proteins of SVV and degrade these viral proteins through the autophagic pathway, inhibiting viral replication of these viral proteins (43). Therefore, we hypothesized that HSPA5 degrades VP2 proteins by binding to them and targeting them to the autophagy pathway. We then further explored the underlying molecular mechanisms. As a major regulator of endoplasmic reticulum stress, HSPA5 plays an important role in the initiation and regulation of autophagy. When cells are faced with protein misfolding or aggregation, HSPA5 induces autophagy by activating the UPR signaling pathway to remove damaged proteins and organelles (17). We therefore hypothesize that HSPA5 degradation of VP2 may be associated with the UPR pathway. HEK-293T cells are widely used to study the unfolded protein response pathway and its associated mechanisms (44). We validated the antiviral effects of HSPA5 using BHK-21 cells. However, previous studies have demonstrated that SVA can replicate in HEK-293T cells (45, 46), and our results further confirm that HSPA5 inhibits SVA proliferation in HEK-293T cells (Fig. S2A). As demonstrated in this study, HSPA5 degradation of VP2 is associated with the activation of the PERK-eIF2α-mediated autophagy pathway, and inhibitor treatment supports the role of the PERK-eIF2α pathway in the degradation of the viral protein VP2 by HSPA5. In the future, we may further validate our findings by overexpressing or knocking out these nodal proteins. Recent studies reveal that VP2 promotes macroautophagy-mediated degradation of PRDX1 (peroxiredoxin 1). It also recruits HSPA8 to specifically target PRDX1 through motif recognition, guiding its degradation via LAMP2A-mediated chaperone-mediated autophagy (CMA), thereby increasing reactive oxygen species (ROS) production to promote viral replication. Clearly, VP2 degradation likely plays a complex role in SVA replication (47).

The HSPA5 functional domains mainly consist of the N-terminal NBD and the C-terminal SBD. In this study, the NBD was also shown to be a key functional domain for the degradation of VP2. Since the ATP hydrolase activity of HSPA5 is entirely mediated by its nucleotide-binding domain (48), we investigated whether this activity influences its degradation of VP2 protein and its interaction with VP2. HA15 is a specific HSPA5 inhibitor that exhibits anticancer activity by targeting and blocking the ATPase activity that mediates chaperone function (49). Our studies revealed that the ATPase activity of HSPA5 is essential for its degradation of VP2 protein and its interaction with VP2 (Fig. S8A through C). Numerous reports have shown that the degradation of proteins is related to the amino acid residues or motifs to which they are anchored. For example, the protease activity of SVA-3C is dependent on a conserved catalytic box with cysteine and cysteine, the cleavage site of severe acute respiratory syndrome coronavirus 2 (SARS-CoV-2) NSP5 usually contains an amide bond (50), and the process of ubiquitination modification is usually associated with lysine residues of the target protein (51). SVA-VP2 T180N was identified as a degradation target of HSPA5. We first examined the impact of the VP2-T180N residue on VP2 protein stability and found no significant difference in protein stability between SVA-VP2 and SVA-T180N (Fig. S3A). Overexpression of HSPA5 significantly accelerated VP2 degradation in CHX-treated cells, indicating that HSPA5 regulates the half-life of SVA-VP2. However, the half-life of the mutant was unaffected, indicating that this site is critical for HSPA5 to accelerate the half-life of the VP2 protein (Fig. S3B). By analyzing the known SVA capsid structure and locating the position of VP2 aa180 within the native structure, we found that this site is exposed externally in the VP2 monomer protein (46) (Fig. S4A through E). We investigated the effect of VP2 T180N on the interaction between VP2 and HSPA5. It was found that this mutation did not disrupt the interaction between VP2 and HSPA5 (Fig. S5A and B). Overexpressed HSPA5 was also able to interact with and colocalize with the VP2 protein in cells infected with the SVA-VP2-T180N mutant recombinant virus (Fig. S1B and D). This indicates that VP2 T180N does not evade degradation by avoiding interaction with HSPA5, suggesting that the interaction site and the site responsible for degradation function are distinct. Resistance of the SVA-VP2 T180N mutant virus to HSPA5-mediated inhibition confirms that this residue plays an irreplaceable role in restricting viral replication. Further analysis of the impact of HSPA5 overexpression on the life cycle of the SVA-VP2-T180N mutant recombinant virus revealed that this mutation enhanced viral replication. We speculate that this effect arises because the VP2 protein is no longer degraded by HSPA5, leading to increased viral particle production during the replication phase (Fig. S6A through E).

**Fig 10:**
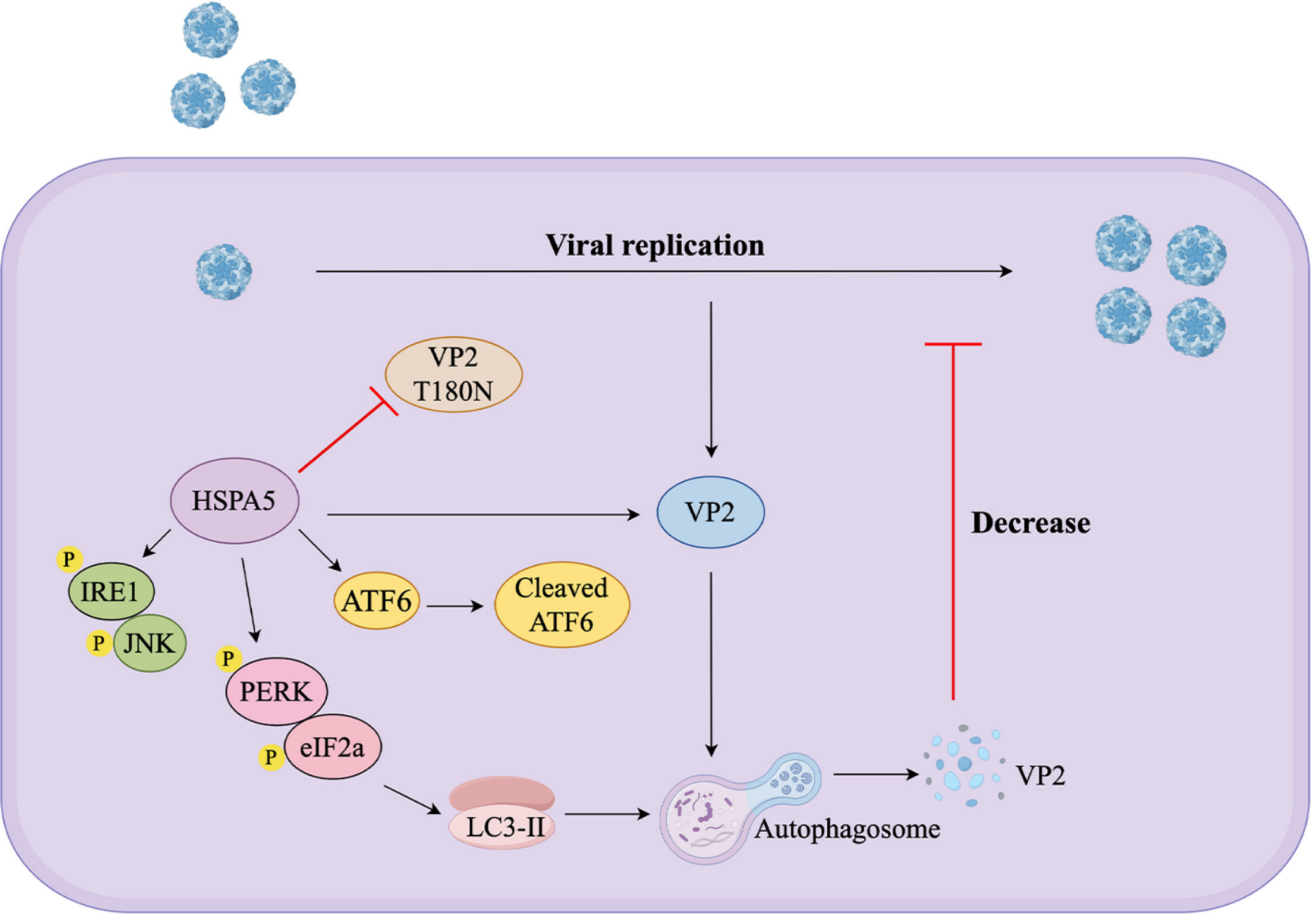
Model of HSPA5-mediated inhibition of SVA replication. HSPA5 inhibits SVA replication by mediating autophagic degradation of the SVA-VP2 protein through activation of the PERK-eIf2α pathway. The T180N mutation in the SVA-VP2 protein results in the inability of HSPA5 to degrade VP2. In addition, VP2-T180R mutant recombinant viruses exhibit resistance to the antiviral activity of HSPA5.

In summary, our results suggest that HSPA5 plays a novel role in inhibiting SVA replication by mediating autophagic degradation of VP2 protein through activation of the PERK-eIf2α pathway. In addition, the NBD functional domain of HSPA5 is a critical region for degrading VP2, and we also found that the Thr180 mutation disrupted VP2 degradation and enhanced SVA replication. Our findings reveal an important molecular mechanism of HSPA5 resistance to SVA infection(Fig. 10), which deepens our understanding of the host’s natural defense mechanisms and contributes to the development of more effective strategies to control SVA infection.

## MATERIALS AND METHODS

### Cells, viruses, antibodies, and chemical reagents

PK-15 cells, BHK-21 cells, PK-1 cells, and HEK-293T cells were cultured in Dulbecco’s modified Eagle’s medium (DMEM, Gibco, USA) supplemented with 10% fetal bovine serum (FBS, Hyclone), 100 μg/mL penicillin, and streptomycin. All cells were cultured in a 5% CO_2_ humidified incubator at 37°C. The SVA (GenBank accession no. MH779611) strain was donated by the International Joint Collaborative Research Laboratory of the Ministry of Education for Animal Health and Food Safety, College of Veterinary Medicine, Nanjing Agricultural University. The SVA-VP2 polyclonal antibody was prepared and stored in the laboratory, and other antibodies were purchased commercially, including rabbit monoclonal anti-PERK antibody (Abmart; MA8131), rabbit monoclonal anti-p-PERK antibody (Abmart; PS00157), rabbit monoclonal anti-IRE1 antibody (Abmart; TA7651), rabbit monoclonal anti-p-IRE1 antibody (Abmart; T55605), rabbit monoclonal anti-JNK1/2/3 antibody (Abmart; T40073), rabbit monoclonal anti-p-JNK1/2/3 antibody (Abmart; T40074), mouse monoclonal anti-ATF6 antibody (Selleck, F0822), rabbit monoclonal anti-elF2α antibody (Selleck; F0316), rabbit monoclonal anti-p-elF2α antibody (Selleck; F0257), rabbit polyclonal anti-HSPA5 antibody (Abmart; P23089-2), mouse monoclonal anti-HA antibody (Abmart; M20003L), mouse monoclonal anti-Flag antibody (Abmart; M20008L), mouse monoclonal anti-GFP antibody (Abmart; M20004L), mouse monoclonal anti-β-microtubule antibody (Abmart; T55605). Horseradish peroxidase (HRP)-labeled anti-mouse IgG (ZenBio, 511103), horseradish peroxidase (HRP)-labeled anti-rabbit IgG (ZenBio, 511203), and FITC-labeled goat anti-rabbit IgG (Biosharp, BL033A). Chemical reagents were purchased commercially, including 3-MA (3-Methyladenine) (Selleck, S2767), MG132 (Selleck, S2619), Ac-DEVD-CHO (Selleck, S7901), Ceapim-A7 (Selleck, E1099), Salubrinal (Selleck, S2923), 4μ8C (Selleck, S7272), Cycloheximide (MCE, HY-12320), and HA15 (Selleck, S8299).

### Cell viability assay

The effects of various chemical reagents on cell viability were evaluated using a Cell Counting Kit-8 (CCK-8) (Biosharp, BS350B). BHK-21 or HEK-293T cells treated with various concentrations of chemical reagents were processed with the corresponding reagents of the CCK-8 kit, and cell viability was measured at specific time points according to the manufacturer’s protocols.

### Western blotting assay

RIPA (Beyotime, P0013B) and PMSF (biosharp, BL507A-1) are mixed thoroughly in a 100:1 ratio and placed on ice for later use. Wash the cells with pre-chilled PBS, add protein lysate and lyse on ice for 15 min, collect the cell lysate, add an appropriate amount of 5× protein loading buffer (biosharp, BL502B), and boil in boiling water for 15 min. Then, it is separated on a polyacrylamide gel. After the proteins in the samples were transferred to the NC membrane, the NC membrane was blocked with PBST solution containing 5% skimmed milk powder for 1 h at room temperature, then incubated with the corresponding primary antibody at a dilution for 2 h at room temperature, and then, the NC membrane was washed three times with PBST solution for 10 min each. It was then incubated with a 1:10,000 dilution of horseradish peroxidase (HRP)-conjugated secondary antibody for 1 h at room temperature, followed by washing three times with PBST for 10 min each. Finally, proteins on the NC membrane were visualized by an ultrasensitive ECL chemiluminescence detection kit (Tanon).

### Plasmids

The genes encoding the HSP family were amplified from PK-15 cell genomic cDNA and cloned into pCAGGS using primers for each gene in the S1 table. pCAGGS vector that had been modified to contain 3′ FLAG-, HA-tag was kindly provided by Dr. Zhigao Bu at the Harbin Veterinary Research Institute, Chinese Academy of Agricultural Sciences (52). SVA protein L, VP1, VP2, VP3, VP4, 2B, 2C, 3A, 3C, 3D, PX459, and SVA-infected cloning plasmid pCMV-rSVA were donated by the Laboratory of Animal Bacteriology, Nanjing Agricultural University. The truncated pCA-HSPA5-HA and pCA-VP2-Flag constructs were subcloned from the pCA-HSPA5-HA and pCA-VP2-Flag plasmids, respectively. A series of Flag-tagged VP2 protein mutants was generated via site-directed mutagenesis PCR. Plasmids encoding GFP and mCherry genes were maintained in our laboratory. The pCA-VP2-GFP and pCA-HSPA5-mCherry plasmids were constructed via overlap PCR (53).

### Construction of a HSPA5 knockout cell line

Three sgRNA sequences were designed using an online CRISPR design tool (https://www.benchling.com) to target HSPA5, as shown in Table S2. Add a Bbs I restriction site to the 5′ end of the sgRNA. The synthesized sgRNA oligonucleotides were annealed at 95°C for 5 min, and then the temperature was raised from 95°C to 25°C at a rate of 0.1°C/s to obtain double-stranded nucleotide fragments. The annealed fragment was cloned into pX-459 to generate sgRNA-HSPA5-1, sgRNA-HSPA5-2, and sgRNA-HSPA5-3 (54). The recombinant plasmid was transfected with 70% confluent BHK-21 cells in a 12-well plate using Lipofectamine 3000 transfection reagent, with the empty vector as the control; 48 h after transfection, replace the transfection medium with fresh cell culture medium (FBS: 10%, puromycin: 0.2 mg/mL). To isolate single-cell clones, cells are cultured to 50% confluence under puromycin selection, diluted to concentrations of 100 and 10 cells/mL, and aliquoted into wells of 96-well cell culture plates. After approximately 1 week of incubation, colonies formed by single cells are identified. HSPA5 protein knockout was identified by sequencing and western blotting using HSPA5 endogenous antibody, and sequencing of HSPA5 was performed.

### RNA extraction and real-time fluorescence quantitative PCR

Total RNA was extracted from cells using the E.Z.N.A. Total RNA Kit (Omega Bio-Tek, Inc., Norcross, GA, USA) and then reverse-transcribed using the HiScript II First Strand cDNA Synthesis Kit (Vazyme Biotechnology Co., Ltd., Nanjing, China). RT-qPCR was performed using the SuperReal PreMix Plus (SYBR Green) (TIANGEN BIOTECH Co, Ltd.) was used according to the manufacturer’s instructions. β-tubulin was used as the reference gene, and all data are expressed as relative fold change (calculated using the 2-ΔΔCT method). All primers for RT-qPCR are presented in Table S3.

### Co-immunoprecipitation

HEK-293T cells were lysed in RIPA buffer containing PMSF. The lysate was centrifuged at 8,000 rpm for 5 min, and the supernatant was incubated with 20 μL protein A/G agarose beads (MCE, HY-K0230) for 1 h at 4°C. Centrifuge the lysate at 2,500 rpm for 5 min. Collect the supernatant and incubate with 2 µg of the appropriate mouse antibody and 40 µL of protein A/G agarose beads overnight at 4°C on a roller. Sepharose beads were collected by centrifugation, washed five times with 1 mL of lysis buffer, and then suspended in 100 μL of lysis buffer. Finally, whole cell lysates and immunoprecipitates are used for western blot.

### Inhibitor treatment

HEK-293T cells were cultured in 24-well cell culture plates until 70% confluent and transfected with related plasmids for 8 h. Then, MG-132, Ac-DEVD-CHO, 3-MA, Ceapim-A7, Salubrinal, 4μ8C, HA15, DMSO, or ddH_2_O were treated and cultured for 16 h, and the cell proteins were collected for western blot.

### Virus titration

BHK-21 cells cultured in 96-well plates were infected with 10-fold serial dilutions of SVA samples in eight replicates. After 1 h of incubation at 37°C, the medium was replaced with fresh DMEM and incubated at 37°C for 72 h. Viral titers were expressed as TCID_50_, and the TCID_50_ of SVA was calculated using the Karber method.

### Confocal microscopy

Climb slides were pre-placed on 12-well cell culture plates, and digested BHK-21 cells were plated later. The next day, when the cell confluency reached 50%, the plasmids pCAGGS-HSPA5-mCherry and pCAGGS-VP2-GFP were transfected, and the empty vector plasmid was used as a control. After 24 h, perform the following operations: cells were washed three times with PBS and fixed with 4% paraformaldehyde for 15 min at room temperature. Cells were washed three times in PBS and treated with DAPI stain for 10 min at room temperature. After washing, the cover sheet is observed and photographed on the machine.

### Construction of infectious mutant recombinant SVA cDNA clones

pCMV-rSVA was digested with Pac I and Mlu I, and the VP2 mutant fragment was amplified by overlap using the primers in Table S1. The overlap PCR product was ligated to the cleaved pCMV-rSVA and named pCMV rSVA-VP2-T180N. The recombinant viral recovery plasmid was then constructed in the same way and named pCMV rSVA-VP2-T180N (R). To rescue the virus, plasmids pCMV rSVA-VP2-T180N and pCMV rSVA-VP2-T180N (R) were transfected into BHK-21 cells with lipofectamine 3000 transfection reagent when the cell fusion reached 80%. An empty plasmid transfection control group was also set up. The cells were cultured at 37℃ with 5% CO2 and observed day by day. When 80% CPE was observed in the test group, the virus solution was frozen and thawed three times, and the virus was harvested at 12,830 × *g*, centrifuged, and the supernatant was separated and stored at −80℃ for spare use, and named as rT180N, and rT180N (R).

### Statistical analysis

GraphPad Prism 8.0 software was used to statistically analyze the data, and the differences between the groups were compared, and *P* < 0.05 (*) indicated significant differences, and *P* < 0.01 (**) and *P* < 0.001 (***) indicated significant differences.

## Data Availability

All data generated or analyzed during this study are included in this article. All other original data supporting the conclusions of this study can be obtained from the corresponding authors upon reasonable request.

## SUPPLEMENTAL MATERIAL

Supplemental figures jvi.02103-25-s0001.pdf Fig. S1 to S8.

Table S1 jvi.02103-25-s0002.xlsx Primers used to generate HSP family protein expression plasmids, HSPA5 protein expression plasmids, mCherry labeled HSPA5 luminescent plasmids, GFP labeled VP2 luminescent plasmids, HSPA5 truncated forms, VP2 truncated forms, VP2 mutants, pCMV rSVA-VP2-T180N plasmids, and pCMV rSVA-VP2-T180N (R) plasmids.

Table S2 jvi.02103-25-s0003.xlsx Primer for generation of HSPA5-KO cells.

Table S3 jvi.02103-25-s0004.xlsx Primers used for RT-qPCR.

ASM does not own the copyrights to Supplemental Material that may be linked to, or accessed through, an article. The authors have granted ASM a non-exclusive, world-wide license to publish the Supplemental Material files. Please contact the corresponding author directly for reuse.
