# Supplementary material for: HSPA5 induces autophagy targeting VP2 through the PERK-eIF2α signaling pathway to inhibit SVA replication
Source: J Virol. 2026 Apr 13;100(5):e02103-25. doi: 10.1128/jvi.02103-25 (PMC13185552; doi:10.1128/jvi.02103-25)
Supplement: Supplemental figures — Fig. S1 to S8. [file jvi.02103-25-s0001.pdf]

FigS1

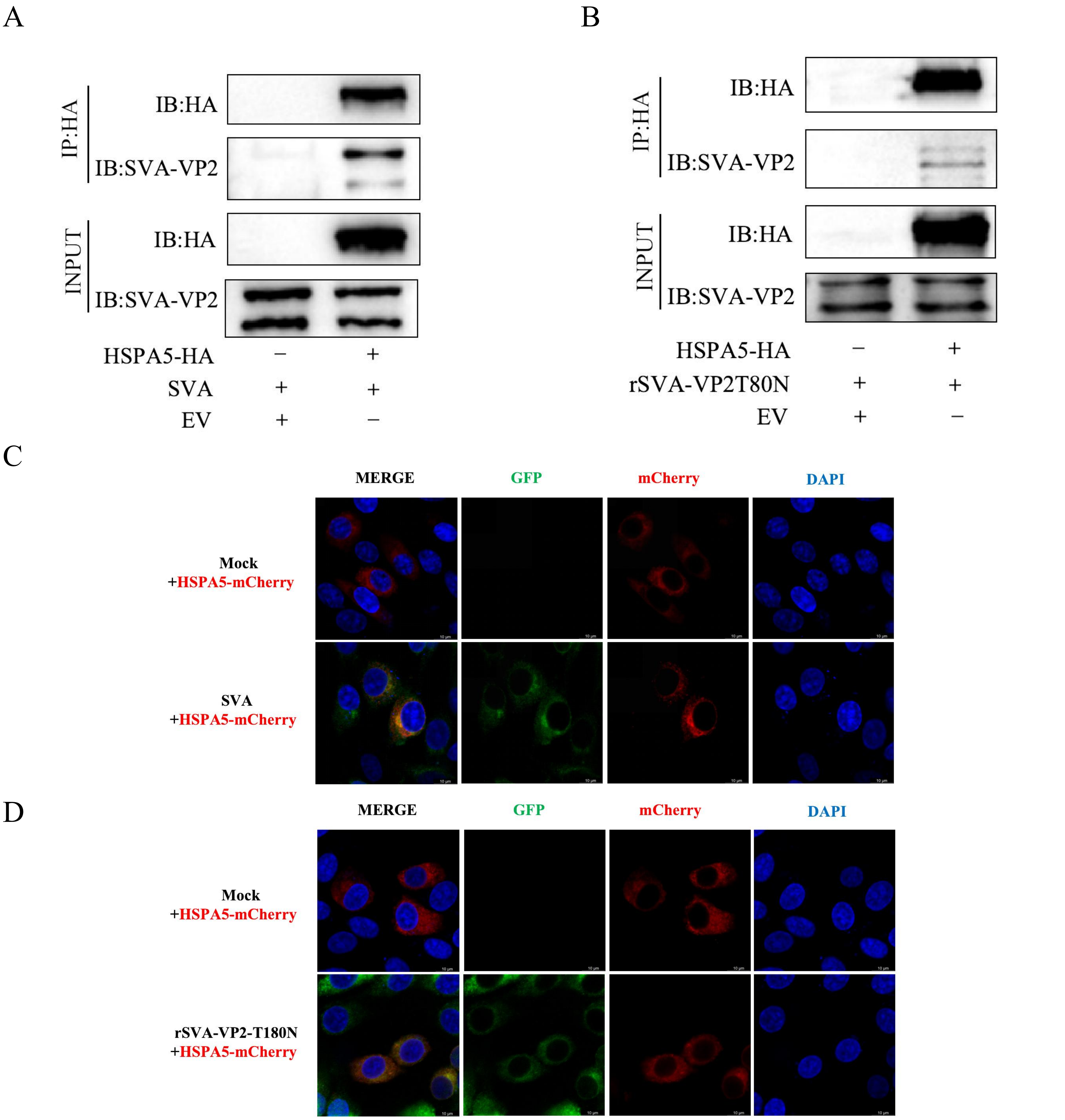

FigS1. HSPA5 interacts with SVA-VP2. (A/B): BHK-21 cells were transfected with either the pCAGGS or pCA-HSPA5-Flag plasmid, and then infected with SVA or rSVA-VP2-T180N (10 MOI) for 16 hours, followed by co-immunoprecipitation (Co-IP) and Western blot analysis using anti-HA antibody. (C/D): Recombinant plasmid pCA-HSPA5-mCherry (red light) were co-transfected into BHK-21 cells , followed by infection with SVA or rSVA-VP2-T180N (1 MOI) for 16 hours, stained with anti-VP2 (green) and DAPI (blue) and observed on confocal laser microscope. Results are from one of three independent experiments.

FigS2

A

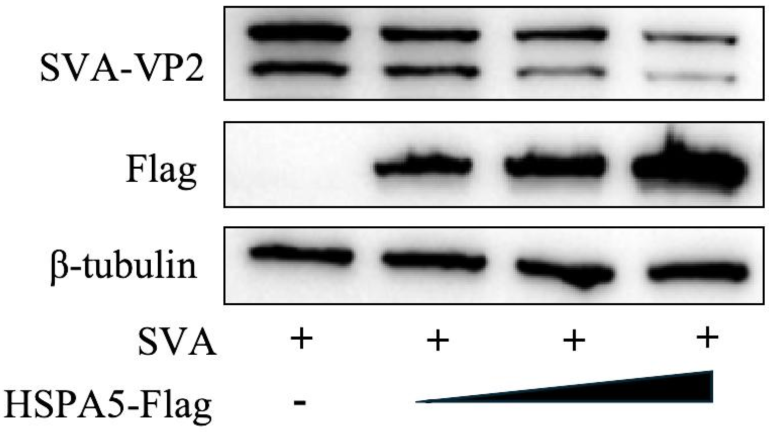

FigS2. Overexpression of HSPA5 in HEK-293T cells inhibits SVA replication in a dose-dependent manner. (A): HEK-293T cells were transfected with different doses of pCA-HSPA5-Flag plasmid, and then infection with SVA (0.01 MOI) for 16 h, the replication of SVA was assessed by western blotting.

FigS3

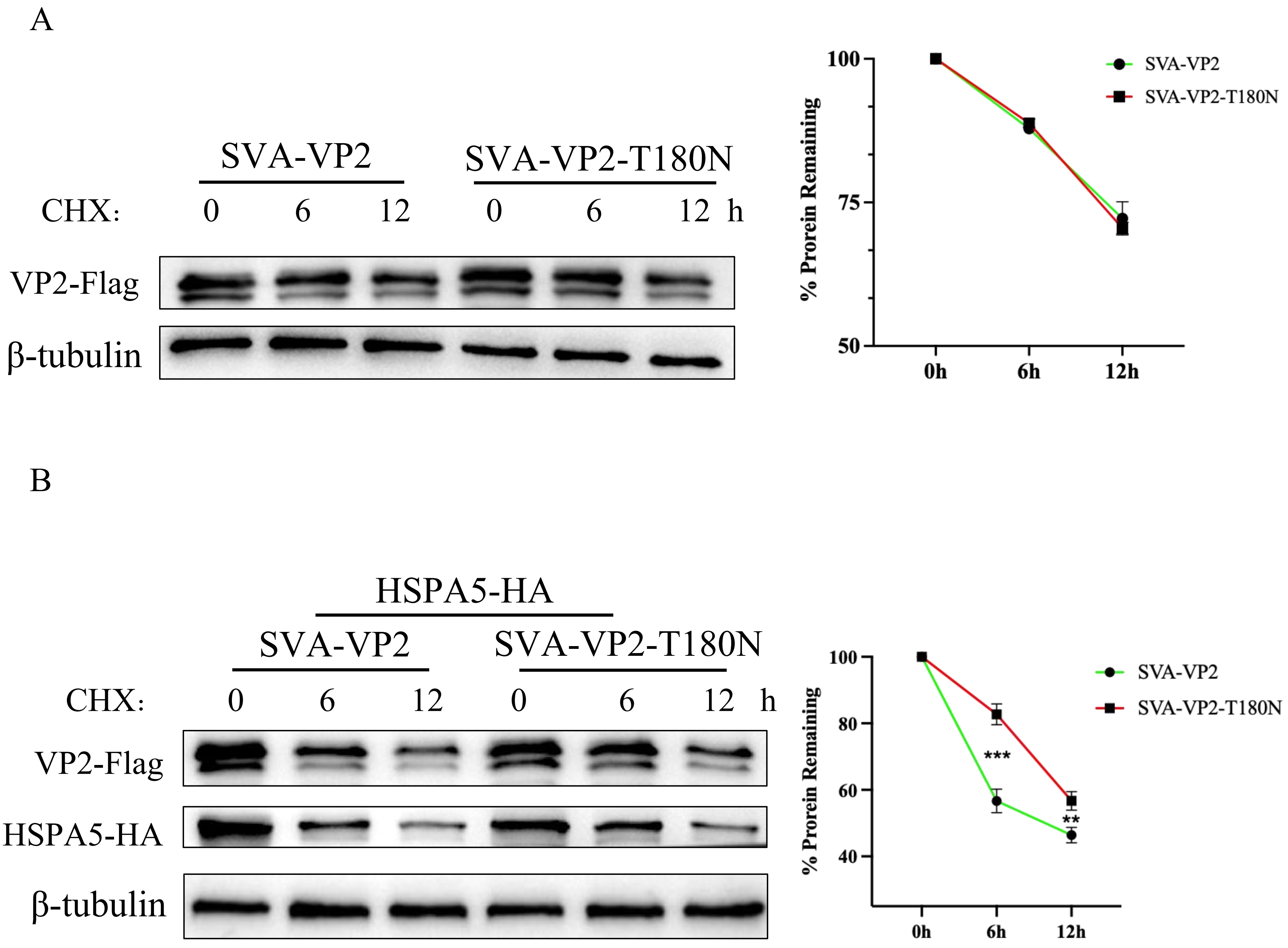

FigS3. No difference in protein stability was observed between SVA-VP2 and SVA-VP2-T180N, with HSPA5 regulating the VP2 protein half-life. (A): pCA-VP2-Flag and pCA-VP2-T180N-Flag plasmids were separately transfected into HEK-293T cells. After 24 hours, cells were treated with chloramphenicol (CHX, 100 μg/ml) followed by Western blot analysis. Relative fold changes in VP2 abundance were determined by densitometry. (B):pCA-VP2-Flag and pCA-VP2-T180N-Flag were co-transfected with pCA-HSPA5-HA into HEK-293T cells. After 24 hours, cells were treated with chloramphenicol (CHX, 100 μg/ml) followed by Western blot analysis. Relative fold changes in VP2 abundance were quantified via density measurement.These results are from one of three independent experiments. Error bars indicate SD. Asterisks in the figure indicate significant differences (***p*<0.01; ****p*<0.001; ns: not significant).

FigS4

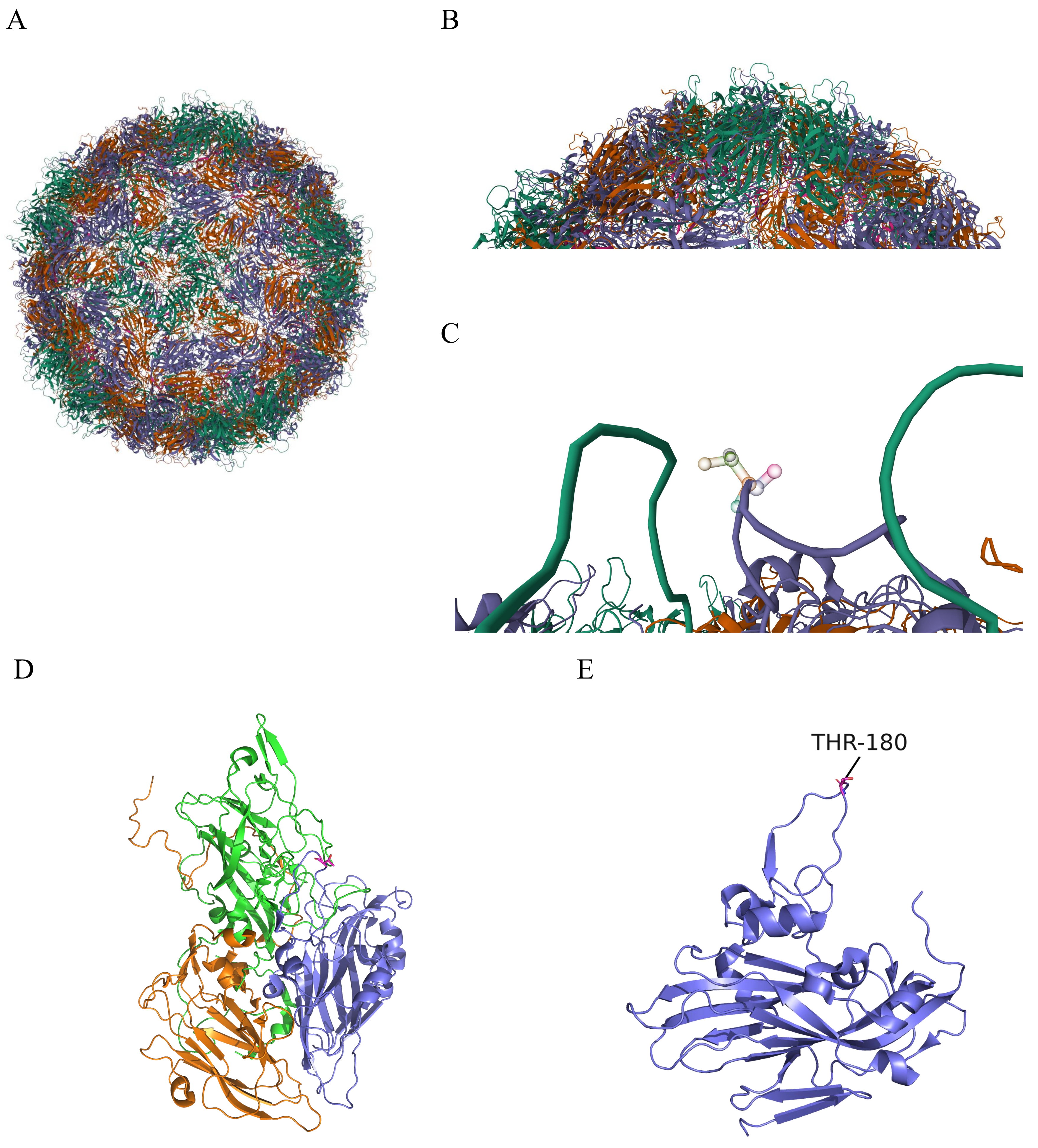

FigS4. The VP2T180 site is located on the exterior of the VP2 protein. (A): This figure displays the atomic-resolution structure (resolution 3.29 Å) of the VP1 (purple), VP2 (green), and VP3 (orange) subunit interface region within a single asymmetric unit of the SVV capsid. (B/C): Ball-and-stick model (multicolored atoms) highlighting the T180 site on the VP2 surface. (D/E): The VP2T180 position is indicated by a purple surface and stick-like structure, located on the exterior of the VP2 subunit.

FigS5

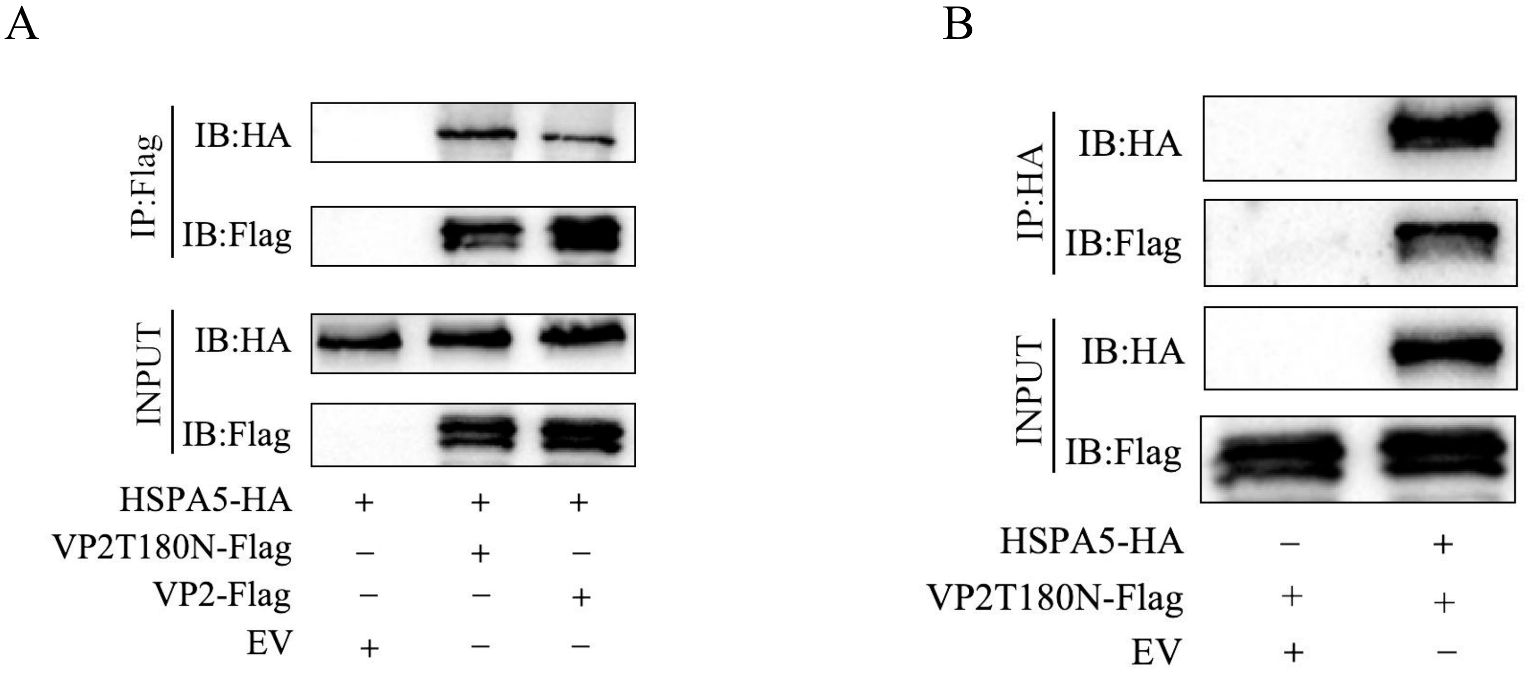

FigS5. HSPA5 interacts with SVA-VP2T80N protein. (A): HEK-293T cells were co-transfected with pCAGGS, pCA-VP2T80N-Flag, and pCA-VP2-Flag respectively, along with pCA-HSPA5-HA. Co-immunoprecipitation (Co-IP) was performed, followed by Western Blot analysis using anti-Flag antibody. (B): HEK-293T cells were co-transfected with pCAGGS, pCA-HSPA5-HA, and pCA-VP2T80N-Flag, followed by co-immunoprecipitation (Co-IP) and Western blot analysis using anti-HA antibody.

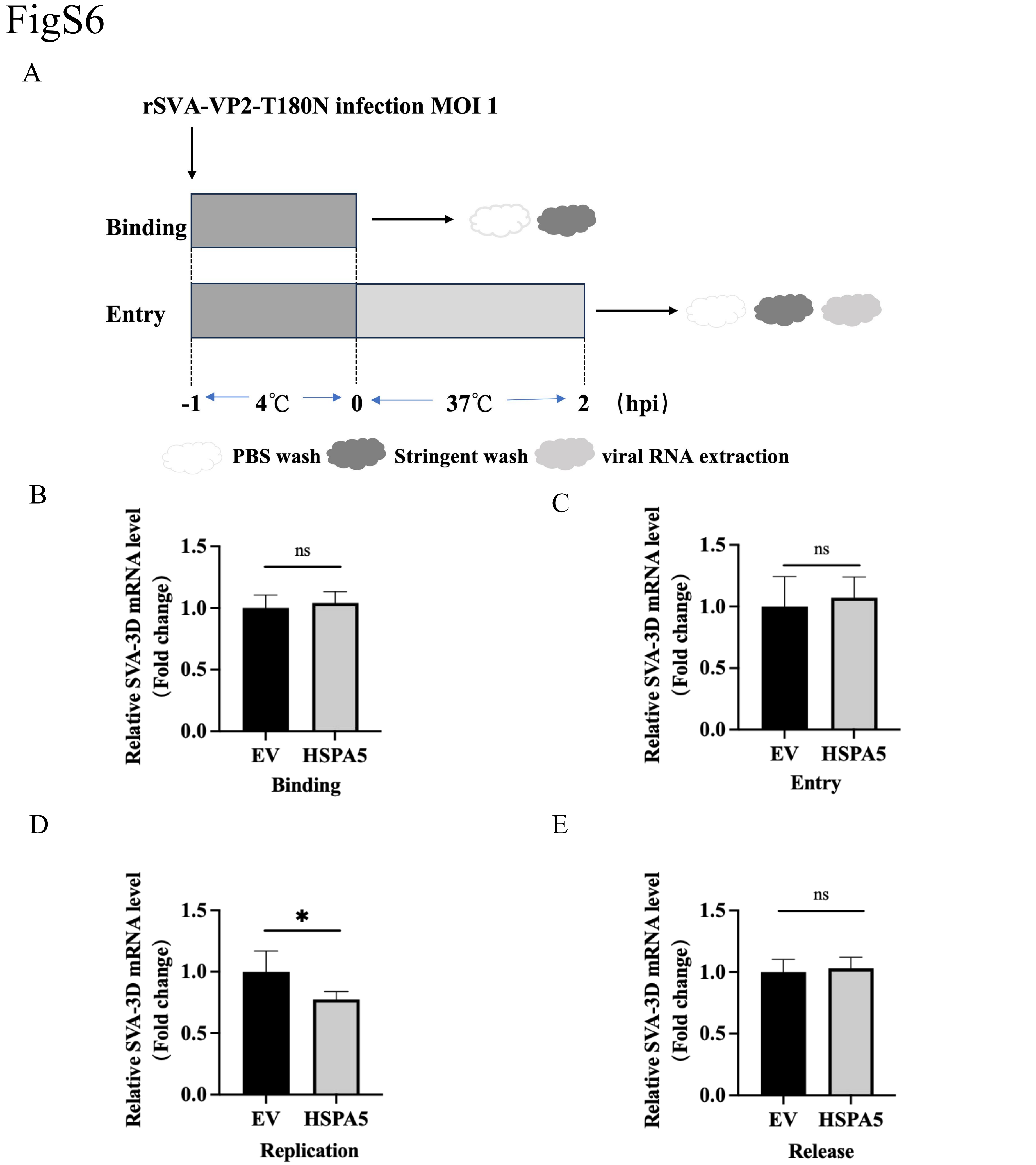

FigS6. Effects of HSPA5 on the rSVA-VP2-T180N life cycle. (A): Overview of the experimental design to examine virus binding and entry. (B/C): qRT-PCR results for viral mRNA (0 and 2 hpi) in pCA-HSPA5-Flag or empty vector-transfected BHK-21 cell challenged with rSVA-VP2-T180N. (D/E): qRT-PCR results for viral mRNA (8 and 16 hpi) in pCA-HSPA5-Flag or empty vector-transfected BHK-21 cells . These results are from one of three independent experiments. Error bars indicate SD. Asterisks in the figure indicate significant differences (**p<0.01; ***p<0.001; ns: not significant).

FigS7

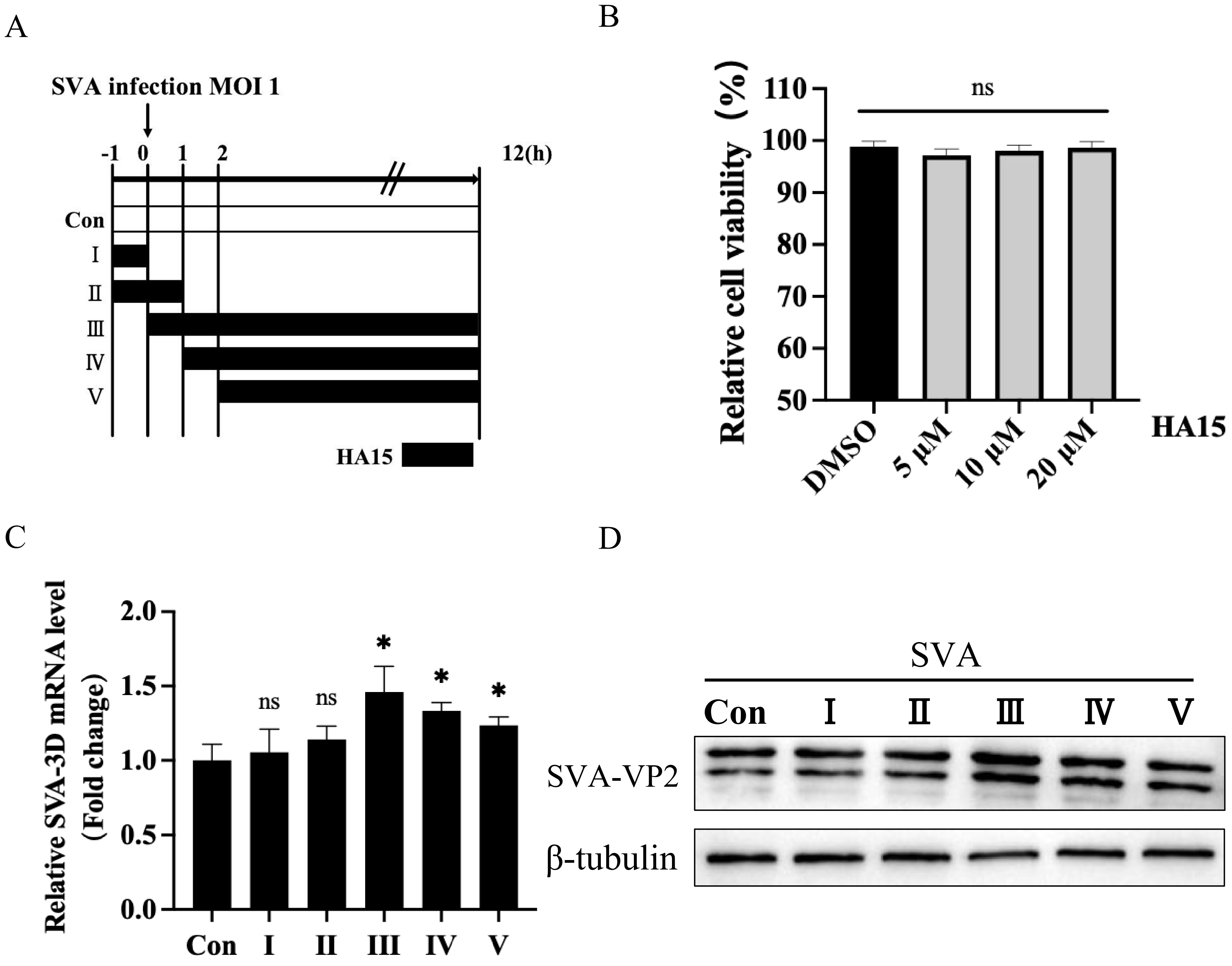

FigS7. HSPA5 participates in the replication phase of the SVA life cycle. (A): Time course diagram of HA15 (10 μM) treatment. The black region represents the HA15-treated group. (B):The detection of cell viability in BHK-21 cells treated with various concentrations of HA15. (C/D): BHK-21 cells infected with SVA at an MOI of 1 were collected at 12 hpi and then analyzed by viral RNA copies (C), and viral VP2 expression (D). These results are from one of three independent experiments. Error bars indicate SD. Asterisks in the figure indicate significant differences (***p*<0.01; ****p*<0.001; ns: not significant).

FigS8

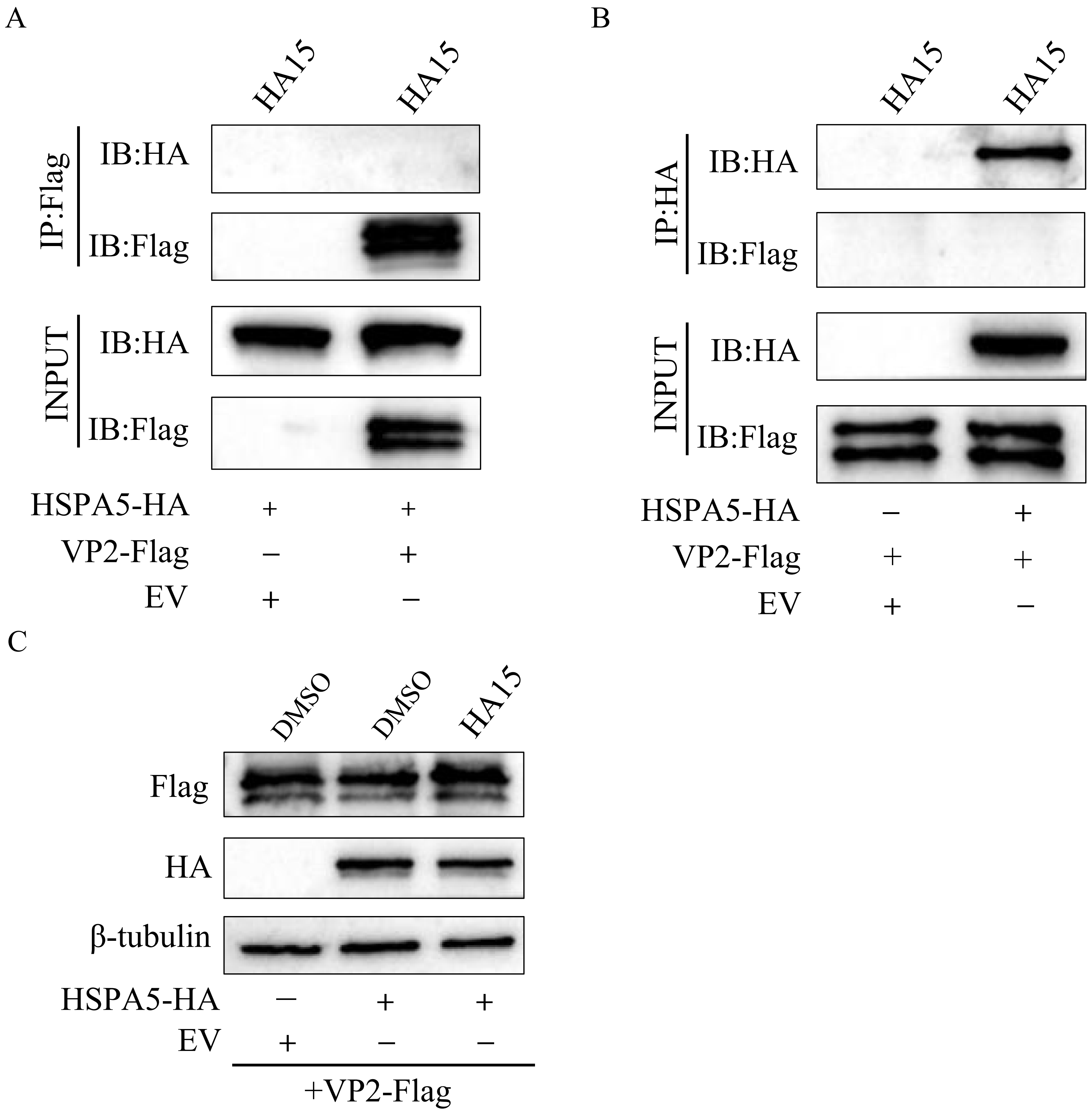

FigS8. The ATPase activity of HSPA5 is essential for its degradation of VP2 protein and interaction with it. (A/B): Co-transfect HEK-293T cells with pCA-VP2-Flag and pCA-HSPA5-HA. Treat cells with HA15 (10 μM) 8 hours post-transfection. Perform co-immunoprecipitation followed by Western blot analysis using anti-Flag and anti-HA antibodies.. (C): HEK-293T cells were transfected with pCA-HSPA5-HA and pCA-VP2-Flag, then cells were treated with HA15 (20 μM) 8 h post transfection. Results are from one of three independent experiments.
